# A Systematic Literature Review on the Global Status of Newborn Screening for Mucopolysaccharidosis II

**DOI:** 10.3390/ijns10040071

**Published:** 2024-10-10

**Authors:** Olulade Ayodele, Daniel Fertek, Obaro Evuarherhe, Csaba Siffel, Jennifer Audi, Karen S. Yee, Barbara K. Burton

**Affiliations:** 1 Takeda Development Center Americas, Inc., Lexington, MA 02421, USA; 2 Takeda Pharmaceuticals International AG, 8152 Zurich, Switzerland; 3 Oxford PharmaGenesis, Oxford OX13 5QJ, UK; 4 College of Allied Health Sciences, Augusta University, Augusta, GA 30912, USA; 5 Takeda Development Center Americas, Inc., Cambridge, MA 02142, USA; 6 Ann & Robert H. Lurie Children’s Hospital of Chicago, Northwestern University, Chicago, IL 60611, USA

**Keywords:** lysosomal storage diseases, mucopolysaccharidosis II, neonatal screening, systematic literature review

## Abstract

A systematic literature review was conducted to determine the global status of newborn screening (NBS) for mucopolysaccharidosis (MPS) II (Hunter syndrome; OMIM 309900). Electronic databases were searched in July 2023 for articles referencing NBS for lysosomal storage diseases: 53 featured MPS II. Until recently, only Taiwan and two US states (Illinois and Missouri) formally screened newborns for MPS II, although pilot programs have been conducted elsewhere (Japan, New York, and Washington). In 2022, MPS II was added to the US Recommended Uniform Screening Panel, with increased uptake of NBS anticipated across the USA. While the overall MPS II birth prevalence, determined from NBS initiatives, was higher than in previous reports, it was lower in the USA (approximately 1 in 73,000 according to recent studies in Illinois and Missouri) than in Asia (approximately 1 in 15,000 in Japan). NBS programs typically rely on tandem mass spectrometry quantification of iduronate-2-sulfatase activity for first-tier testing. Diagnosis is often confirmed via molecular genetic testing and/or biochemical testing but may be complicated by factors such as pseudodeficiency alleles and variants of unknown significance. Evidence relating to MPS II NBS is lacking outside Taiwan and the USA. Although broad benefits of NBS are recognized, few studies specifically explored the perspectives of families of children with MPS II.

## 1. Introduction

Lysosomal storage diseases (LSDs) are a group of more than 50 inherited metabolic conditions [1], typically resulting from enzyme deficiencies that interfere with lysosome function. Mucopolysaccharidosis (MPS) II (Hunter syndrome; OMIM 309900) is a progressive, X-linked LSD caused by variants in the iduronate-2-sulfatase (I2S) gene (*IDS*), which results in deficiency of the I2S enzyme required for the lysosomal degradation of glycosaminoglycans (GAGs), such as heparan sulfate (HS) and dermatan sulfate (DS) [2]. This leads to lysosomal GAG accumulation, resulting in multisystemic cell and organ dysfunction, significant morbidity, and shortened life expectancy. MPS II displays a broad spectrum of severity. Associated somatic signs and symptoms (such as coarse facial features and joint and skeletal abnormalities) may be accompanied by cognitive impairment in approximately two-thirds of patients [2,3]. MPS II predominantly affects individuals of male sex and occurs in 1.3 in 100,000 to 1 in 165,000 live births [4,5]. Although MPS II pathology can be evident during fetal development, infants are often developmentally normal at birth [6]. Diagnostic delays are common owing to the rarity of the disease and because early signs and symptoms (such as ear, nose, and throat abnormalities and hernias) are also common in the general pediatric population and often attributed to other causes [7]. Additionally, although some signs and symptoms of MPS II present in late childhood or adulthood, most of them frequently emerge in the first years of life [8]. In a patient with suspected MPS II, analysis of urinary GAGs is typically conducted as an initial screening assay for increased levels of HS and DS. If present, the diagnosis is confirmed by demonstrating deficiency of I2S enzyme activity in leukocytes, fibroblasts, or plasma. Genetic analysis of the *IDS* gene may also be utilized to support or confirm a diagnosis in some cases [9]. MPS II is typically caused by point mutations or small insertions or deletions; however, approximately 20% of patients have large *IDS* deletions or major gene rearrangements caused by homologous recombination between the functional *IDS* gene and an *IDS* pseudogene [10]. However, genetic analysis of *IDS* can be complicated by the identification of variants of unknown significance (VUS) and pseudodeficiency alleles, which result in low I2S enzyme activity but do not cause disease [11].

Intravenous enzyme replacement therapy (ERT) with recombinant idursulfase (Elaprase, Takeda Pharmaceuticals USA., Inc., Lexington, MA, USA) has been available since 2006 and is the recommended treatment for MPS II [9,12,13,14]. Intravenous idursulfase has been shown to stabilize or to improve several clinical parameters, including walking capacity, lung function, cardiac hypertrophy, liver and spleen sizes, and joint function [9,15,16]. Additionally, analysis of long-term real-world data has shown increased survival rates in patients with MPS II treated with ERT compared with untreated patients [17]. Consistent with the clear relationship between progressive GAG storage and clinical manifestations in MPS II, clinical guidelines/recommendations for the management of MPS II make a clear case for the initiation of ERT as early as possible after diagnosis [9,18]. The need for early diagnosis to facilitate optimal treatment outcomes is well accepted [19,20]. As ERT is unable to cross the blood–brain barrier, patients may receive hematopoietic stem cell transplantation (HSCT) to treat the neurological symptoms; however, the use of HSCT in patients with MPS II is controversial and it is only available in some countries [11]. Additionally, adaptations to ERT to facilitate crossing the blood–brain barrier are under investigation, including the use of intracerebroventricular or intrathecal administration routes or the creation of modified fusion proteins, as well as novel therapies such as gene therapy and gene editing [11]. Such approaches would likely also benefit from the early diagnosis of patients with MPS II.

The importance of early diagnosis is not restricted to MPS II but is relevant to many LSDs, prompting their incorporation within newborn screening (NBS) programs in some regions in North and South America [21,22], Asia [23,24,25], and Europe [26]. Advances in screening methods have addressed many technical concerns regarding NBS, but challenges remain, including deciding which LSDs should be included in routine screenings. To date, specific screening for MPS II has had limited integration into NBS programs at national levels. The main exception to this is Taiwan, where pilot NBS programs have been progressively implemented since 2015 [27]. In August 2022, MPS II was added to the Recommended Uniform Screening Panel (RUSP), the list of conditions recommended for NBS in the USA, after approval by the US Department of Health and Human Services [28,29]. This means that, over time, most US states (those that have pledged to abide by recommendations from the Advisory Committee on Heritable Disorders in Newborns and Children) can now be expected to move towards implementing NBS for MPS II [30].

Based on a comprehensive systematic literature review (SLR), we illustrate the global status of NBS for MPS II and identify some of the challenges that exist, such as procedural or technical barriers that impede implementation of NBS programs. We also consider the impact such programs have on estimated disease incidence and prevalence, mortality and morbidity outcomes, and patient follow-up and management and detail the technological developments that may facilitate implementation of NBS for MPS II.

## 2. Methods

### 2.1. Search Strategy

The purpose of this study was to identify literature relating to NBS for MPS II, with specific topics of interest including the status of MPS II NBS programs worldwide, technologies and methodologies employed, and outcomes of established programs and pilot studies. Barriers to the implementation of NBS programs, the impact of screening and diagnosis on disease management, and future considerations for recommending NBS were also investigated.

Publications were identified by systematic electronic searches of Embase (1974–present), MEDLINE (In-Process & Other Non-Indexed Citations and Ovid MEDLINE (1946–present)), and the Cochrane Library (Cochrane Database of Systematic Reviews; Database of Abstracts of Reviews of Effects; Cochrane Central Register of Controlled Trials; Cochrane Methodology Register; NHS Economic Evaluation Database; Health Technology Assessment Database; American College of Physicians Journal Club). Electronic database searches were run on 10 August 2021 and 3 July 2023. Search results were downloaded into EndNote X9 (Clarivate Analytics, Philadelphia, PA, USA) and deduplicated before eligibility screening. These searches were supplemented with manual searches of relevant congress proceedings from 2019 to 2023 (the Lysosomal Disease Network Annual WORLD*Symposium*, the Annual Meeting of the Society for the Study of Inborn Errors of Metabolism, and the MPS Society International Symposium on MPS and Related Diseases).

In the initial search (August 2021), a broad strategy was used to identify publications reporting NBS for LSDs in general, which may also have pertinent information on MPS II. In the search update (July 2023), non-MPS II-specific publications were excluded. Search strings were combinations of free text and Medical Subject Headings terms relating to NBS, LSDs, and MPS II (Supplementary Table S1). This SLR was conducted in accordance with the 2020 Preferred Reporting Items for Systematic Reviews and Meta-Analyses (PRISMA) guidelines [31], and the protocol was registered with the international Prospective Register of Systematic Reviews (PROSPERO; CRD42022288220) [32].

### 2.2. Publication Eligibility

The title and abstracts of all publications identified in the electronic searches underwent double-blind screening by two independent reviewers against the predefined eligibility criteria (Supplementary Table S2) to ensure all relevant evidence was captured and to minimize bias. Studies of patients undergoing NBS for any LSD (initial search) or MPS II (updated search), without restriction by intervention, comparator, study design, date, or country were eligible for inclusion (see Supplementary Table S2 for full eligibility criteria). Reasons for study selection were categorized, and reasons for exclusion were chosen from a prespecified list. Full-text articles meeting the inclusion criteria were screened to confirm eligibility and shortlisted for data extraction (Supplementary Table S2). Title/abstract screening and full-text review were conducted by two reviewers in parallel, with discrepant opinions resolved by discussion or escalation to a third reviewer.

### 2.3. Data Extraction

One or two reviewers extracted all information from the eligible publications in parallel; all extracted data were subjected to quality control by an additional reviewer. The following information was extracted from each eligible publication: author(s); reference details; title; year of publication; type of article; study design; type of LSD (initial search only); key findings, including details of NBS program or pilot study, target LSD population (initial search only), and definition used; methodologies/technologies used; diagnostic testing validity and reliability assessments; impact on mortality and morbidity, incidence, and prevalence of LSDs (initial search only)/MPS II (updated search only); patient follow-up, management, and treatment of patients; challenges with NBS methods; patient identification and implementation; and future considerations.

## 3. Results

### 3.1. Included Studies

The screening process for identification and shortlisting of publications is summarized in the PRISMA flow chart (Supplementary Figure S1).

In total, the searches identified 53 articles that covered the topics of interest in MPS II (Supplementary Table S3): 38 full-text articles [22,23,25,27,29,30,33,34,35,36,37,38,39,40,41,42,43,44,45,46,47,48,49,50,51,52,53,54,55,56,57,58,59,60,61,62,63,64,65] and 15 congress abstracts [66,67,68,69,70,71,72,73,74,75,76,77,78,79].

Among the fifty-three MPS II publications, there were forty-two original reports based on laboratory studies focusing on the development or validation of screening assays and methodologies and/or reporting the results from NBS studies, four narrative reviews, two SLRs, one combined narrative review and survey, one evaluative analysis, one cross-sectional study, one prospective observational study, and one survey of healthcare professionals (HCPs). Most publications (40 of 53) also reported data on other types of MPS and/or other LSDs. When categorized according to topics of interest, 21 articles described the status of NBS programs for MPS II, 44 articles reported various technologies used in screening for MPS II, and 17 articles reported outcomes relating to the estimated birth prevalence of MPS II. In addition, six articles reported the follow-up and outcomes of patients identified as having MPS II through NBS and the challenges associated with implementing NBS programs for MPS II. Notably, one of these articles assessed the readiness of NBS programs in the USA for implementing comprehensive screening for MPS II after its inclusion in the RUSP and likely influencing factors [29].

### 3.2. Status of NBS Programs for MPS II and Estimated Birth Prevalence Based on NBS

NBS programs that included MPS II and that had been established by the time of the database search (July 2023) were identified in Taiwan [23,27,39,41,53,67] and in Illinois and Missouri in the USA [29,30,34,36,37,38,73]. In addition, pilot studies for MPS II NBS are being carried out in the US states of Washington [22] and New York [77] and in Japan (Table 1) [25,79].

Seventeen articles reported MPS II birth prevalence in Taiwan [23,27,39,41,53,66,67], Japan [25,79], and the USA [22,30,34,36,37,38,47,71] (Table 2). In Taiwan, when the routine NBS program for MPS was established in 2015 [23], the birth prevalence of MPS II ranged from 1 in 24,581 to 1 in 60,671 [23,27,39,41,67]. For context, the national birth rate in Taiwan is approximately 200,000 babies per year [80,81]. The birth prevalence of MPS II in Japan (where approximately 800,000 babies are born each year [81]) was reported to be 1 in 18,222 based on screening of samples between 2012 and 2015 [25]. Data from the pilot NBS program in Tokyo metropolitan and surrounding areas revealed two cases in more than 30,000 screened newborns [79]. Together, these studies suggest a higher birth prevalence of MPS II in Taiwan and Japan than in the USA. A narrative review addressing the inclusion of MPS II in the RUSP reported that the estimated birth prevalence of MPS II ranged from 1 in 100,000 to 1 in 150,000 in the USA [30].

In Missouri (70,000 births in 2024 [82]), prospective NBS for MPS II began in November 2018. The birth prevalence of MPS II was estimated to be approximately 1 in 60,000 births, after screening approximately 150,000 newborns over an 18-month period (to June 2020) from the state NBS program [34]. In December 2017, Illinois became the first US state to implement population-based NBS for MPS II. In this state with ~132,000 births in 2021 [83], the birth prevalence was estimated to be 1 in 162,000 from the first report of the NBS program incorporating MPS II screening (2017–2018) [36]; this rate increased as the number of newborns screened increased and was updated to 1 in 113,090 after screening 339,269 infants (2017–2020) and to 1 in 73,290 after screening 586,323 infants (2017–2022) [36,37,38]. In the pilot study of 100,000 samples carried out in Washington in 2019 (80,000 births in 2020), the estimated MPS II birth prevalence was 1 in 105,214 infants [22].

**Table 2:** MPS II epidemiology findings.

Region, Year	Technology	Cutoff Values	False Positive Rate	Estimated Birth Prevalence
Japan, 2020 [25]	DBS GAG (DS and HS) quantification by LC–MS/MS DBS I2S activity by fluorometric assay	>23 ng/mL for HS-NS, >88 ng/mL for DS, and/or >90 ng/mL for HS-0S	1.64% ^a^	1/18,222
Japan, Tokyo, 2022 [79]	Quantification of lysosomal enzyme activity in urine samples by LC–MS/MS	NR	NR	~1/15,000 ^b^
Taiwan, 2019 [39]	DBS I2S activity by multiplex MS/MS Gene sequencing	30% of mean I2S activity, 6.5 μmol/L/h	0.18% ^c^	1/43,391
Taiwan, 2019 [67]	DBS I2S activity by MS/MS Gene sequencing	Initial cutoff: 6.5 μmol/L/h I2S activity Retest cutoff: 2.2 μmol/L/h I2S activity ^d^	0.048% ^e^	1/51,000 ^e^
Taiwan, 2020 [23,66]	DBS I2S activity by multiplex LC–MS/MS Gene sequencing	≤5% of normal mean I2S activity	0.039% ^f^	1/24,581
Taiwan, 2021 [27]	Enzyme activity by MS/MS Total GAG quantification by DMB/Cre ratio GAG-derived disaccharide (CS, DS, HS, and KS) quantification by MS/MS Leukocyte enzyme activity by fluorometric assay Molecular DNA analysis	Normal reference value for DMB/Cre ratio for infants aged <6 months, <70.68 mg/mmol Cre (mean [2 SDs], 41.83 [28.85] mg/mmol Cre) >0.80 ng/mL for DS, <0.78 ng/mL for HS, >7.90 ng/mL for KS Individual enzyme activity ~5% lower than normal, defined as marked reduction	0.06% ^g^	1/34,192 ^g^
Taiwan, 2022 [41]	Mutant gene expressions of enzyme activity by in vitro COS-7 cell transfection assay Quantification of urinary GAG-derived disaccharides (CS, DS, HS, and KS) by MS/MS	Normal reference value for DMB/Cre ratio for infants aged <6 months, <70.68 mg/mmol Cre (mean [2 SDs], 41.83 [28.85] mg/mmol Cre)	0.04% ^h^	1/60,671 ^h^
Taiwan, 2022 [53]	I2S activity by MS/MS Leukocyte I2S enzyme activity by fluorometric assay Total GAG quantification by DMB/Cre ratio GAG-derived disaccharide (CS, DS, HS, and KS) quantification by MS/MS Nucleotide variation detected by Sanger sequencing I2S activity in extracts of COS-7 cells expressing I2S for mutant cDNAs	First DBS cutoff value: 6.5 μmol/L/h (based on 30% of mean I2S activity) Second DBS cutoff value: 2.2 μmol/L/h (based on 10% of mean I2S activity) Marked reduction in I2S activity (reference range 12.89–131.83 μmol/g protein/4 h) <0.80 μg/mL for DS, <0.78 μg/mL for HS, <7.90 μg/mL for KS	<0.50%	NR
USA, California, 2014 [71]	Gene sequencing	NR	NR	1/3500 ^i^
USA, Illinois, 2019 [36]	DBS I2S activity by MS/MS Gene sequencing	Initial cutoff: ≤10% of daily median I2S activity Retest cutoff: >10–13% of daily median I2S activity If retest borderline or positive, referred for diagnostic testing	0.009% ^j^	1/162,000
USA, Illinois, 2020 [37]	DBS I2S activity by MS/MS Gene sequencing	Initial cutoff: ≤10% of daily median I2S activity Retest cutoff: >10–13% of daily median I2S activity If retest borderline or positive, referred for diagnostic testing	0.007% ^k^	1/113,090
USA, Illinois, 2023 [38]	DBS I2S activity Total GAGs analysis using DMB incorporation and spectrophotometry GAG-derived disaccharide (HS and DS) quantification by ESI–MS/MS Molecular testing	Initial cutoff: ≤10% of daily median I2S activity Retest cutoff: >10–13% of daily median I2S activity If retest borderline or positive, referred for diagnostic testing	NR	1/73,290
USA, Missouri, 2020 [34]	First-tier screening: DBS I2S activity by fluorometric assay Second-tier screening: gene sequencing/GAG analysis of DBS	Retest cutoff: 40 μmol/L/h I2S activity High-risk cutoff: 35 μmol/L/h I2S activity	0.018% ^l^	1/73,477
USA, Washington, 2020 [22]	I2S activity by multiplex LC–MS/MS	I2S activity <10% of daily mean	0.016% ^m^	1/105,214
USA, 2018 [47]	NA	NA	NA	1/100,000–170,000 ^n^
USA, 2022 [30]	First-tier screening: DBS I2S activity by MS/MS or microfluorometry Second-tier screening: molecular and/or biochemical testing	NR	NR	1/100,000–150,000 ^o^

^a^ 300 of 18,222 samples tested positive in the first screen, with one confirmed positive diagnosis of MPS II. ^b^ 2 of approximately 30,000 newborns who received a diagnosis of MPS II. ^c^ 240 of 130,175 samples had I2S activity ≤30% of the normal mean, with three confirmed positive diagnoses of MPS II. ^d^ I2S activity measured before referral for biochemistry examinations in study. ^e^ 76 of 153,032 samples had GAG levels below initial cutoff values, with three confirmed positive diagnoses of MPS II. ^f^ 32 of 73,743 samples were found to have I2S activity ≤5% of the normal mean, with three confirmed positive diagnoses for MPS II. ^g^ 186 of 307,731 samples with suspected MPS II found to have reduced enzyme activity were referred for confirmatory testing, with nine confirmed diagnoses of MPS II. ^h^ 223 of 546,040 infants were referred for confirmation, with nine confirmed positive diagnoses of MPS II. ^i^ MPS II unconfirmed; the authors acknowledge that this is an unexpected finding. ^j^ 16 of 162,000 samples were found to have I2S activity ≤10% of the daily median, with one confirmed positive diagnosis of MPS II. ^k^ 28 of 339,269 samples were found to have I2S activity ≤10% of the daily median, with three confirmed positive diagnoses of MPS II. ^l^ 29 of 149,954 samples had an I2S activity of <35 μmol/L/h and were sent for second-tier testing, with two confirmed positive diagnoses of MPS II. ^m^ 18 of 105,214 samples had I2S activity <10% of the daily mean, with one confirmed positive diagnosis of MPS II. ^n^ Range of estimates from MPS II-related websites [84,85]. ^o^ Unclear how estimates were formulated. cDNA, complementary DNA; Cre, creatinine; CS, chondroitin sulfate; DBS, dried blood spot(s); DMB, dimethylmethylene blue; DS, dermatan sulfate; ESI, electrospray isotope dilution; GAG, glycosaminoglycan; HS, heparan sulfate; HS-0S, heparan ΔDi-0S [2-acetamido-2-deoxy-4-O-(4-deoxy-α-L-*threo*-hex-4-enopyranosyluronic acid)-D-glucose]; HS-NS, heparan ΔDi-NS [2-deoxy-2-sulfamino 4-O-(4-deoxy-α-L-*threo*-hex-4-enopyranosyluronic acid)-D-glucose]; I2S, iduronate-2-sulfatase; KS, keratan sulfate; LC, liquid chromatography; MPS, mucopolysaccharidosis; MS/MS, tandem mass spectrometry; NA, not applicable; NBS, newborn screening; NR, not reported.

### 3.3. Technologies Used in NBS for MPS II

A total of 44 identified articles described the technologies used to perform NBS for MPS II (Table 3). As a first-tier screen, systematic MPS II NBS programs used tandem mass spectrometry (MS/MS) (Taiwan and Illinois) [23,27,36,37,38,39,41,53,73], liquid chromatography (LC)–MS/MS (Japan) [79], or fluorometry (Missouri) [34] to measure reduced I2S activity in dried blood spots (DBS). Fluorometric I2S assays are an increasingly popular alternative to MS/MS, due in part to fluorometers being less expensive, more widely available in public health laboratories, and easier to use than mass spectrometers. Some of the established programs also employed additional testing using GAG levels or molecular sequencing: in Missouri [34], GAG level analysis or molecular sequencing was performed after a positive first-tier test result to reduce the rate of false positive screens. In the Taiwanese program [39], genotyping was performed for samples with consistent below-cutoff enzyme activity measurements. Positive screens from NBS programs were then referred for diagnostic testing in contracted follow-up centers outside of the NBS programs. Various evaluations have investigated the use of other assays and techniques for potential first-tier screening for MPS II, including fluorometric enzyme testing of DBS using digital microfluidics, which is an attractive candidate for high-throughput NBS [46,75,86,87,88]. Some laboratories have employed measurement of GAGs (in DBS or (rarely) wet blood samples, in urine using dimethylmethylene blue/creatinine (DMB/Cre) ratio, or quantification of urinary GAG-derived disaccharides by MS/MS) for first-tier screening [45,59,63,74,76,89] or for diagnostic confirmation after measurement of enzyme activity [37,38,41,42,53].

In Taiwan, cutoffs for I2S activity in DBS obtained in 2015 were 30% of the mean (or 6.5 μmol/L/h). In 2016, the retest cutoff for the second DBS was reduced from 30% to 10%, based on the early experience gained in 2015 [39]. This resulted in the number of recalled cases falling from 1.2% to 0.5%, although the authors did not provide further rationale for the change in the cutoff threshold. With a cutoff of 10% of the mean I2S activity for both the first and second DBS, the recall rate was 102 per 100,000 and the positive rate (number needed to be referred) was 49 per 100,000 DBS samples [39]. A report on the NBS program in Taiwan reports use of the 30% and 10% cutoff values for the first and second DBS, respectively, with 41 per 100,000 referred [41]. The NBS program in Illinois and the pilot study in Washington both used a cutoff of 10%, and 76 of 586,323 samples [38] and 28 of 339,000 samples [37], respectively, were positive.

Although not essential for confirmation of diagnosis, genotyping may be performed after first-tier screening in contracted centers to aid in prediction of phenotype and for purposes of genetic counseling. This approach is often used either as part of multistage testing to increase accuracy and to reduce false positives [27,34,39] or as a diagnostic testing strategy after referral [23,36,37,38,53]. Using *IDS* sequencing to identify pathogenic MPS II genotypes, false positive rates were lower in the USA (0.007–0.018%; four studies) [22,34,36,37] than in Japan (1.64%; one study) [25] or Taiwan (0.039–<0.50%; six studies) [27,39,41,53,66,67]. In the majority of cases, false positives were attributed to variants presumed to be pseudodeficient or VUS [34,37,39] and led to the conclusion that the prevalence of presumed pseudodeficiency for I2S is more common than true deficiency [37]. Although they appeared to occur in all racial and ethnic groups, some specific pseudodeficiency alleles seemed to be common in Asian populations [37]. In patients with VUS without a clear diagnosis, there is a need for regular follow-up [34,39].

### 3.4. MPS II NBS Implementation and Outcomes

No studies were identified that reported the impact of NBS programs on morbidity and mortality in patients with MPS II; however, six articles reported research on the implementation of NBS for MPS II, including the follow-up and outcomes of patients with MPS II identified through NBS programs [29,47,48,53,54,65].

In their narrative review, Yamaguchi et al. (2008) described changes in NBS in Japan since 1977. Neonatal screening was described as being affected by national economic instability, a declining birth rate, the development of screening technologies (namely MS/MS), and the advent of treatments such as ERT for MPS, which had influenced the potentially eligible conditions for consideration within NBS panels [65]. The authors highlighted the need for NBS to be as effective and cost-efficient as possible, which remains a key consideration.

More recently, Joseph et al. (2018) conducted an SLR to identify evidence answering the research question, “what should nurses know about MPS II to offer the best care for families who manage a child with MPS II at home?” One aspect discussed was NBS, although the authors mainly reference articles of disease management rather than NBS for MPS II per se [47]. A key finding from the review was the importance of early disease detection and early treatment initiation for the successful management of MPS II. Thus, the authors encouraged HCPs to advocate for MPS II NBS with the goal to delay disease progression. The authors also concluded that families of patients with MPS II should be offered genetic counseling and support services and that more research is essential to identify the long-term effects of MPS II on families.

Collecting insights directly from stakeholders, Lisi et al. conducted a telephone survey in 2013 of 38 genetic HCPs (medical geneticists board-certified in biochemical or general genetics, genetic counselors who work in metabolic or LSD clinics, and directors of biochemical genetics laboratories) regarding population-based NBS for LSDs, including MPS II [54]. Overall, HCPs generally favored NBS for diseases having an available treatment with proven efficacy, and diseases with available ERTs were most positively considered: focusing on LSDs, on average, survey participants favored NBS for MPS II ahead of NBS for Gaucher, Fabry, and Krabbe diseases, and after NBS for Pompe disease and MPS I. This “ranking” is probably driven by the availability of different treatment options, given that, when asked to rate factors that support including a disease on NBS panels, more than 70% of HCPs ranked availability of treatment as being very important or important. In addition, the survey identified the need to support parents whose child is identified as having a VUS and to ensure adequate resources for patient follow-up after a positive diagnosis [54].

Jones et al. (2022) conducted a comprehensive assessment of 48 inherited metabolic diseases using the inherited metabolic diseases NBS evaluation algorithm [48]. This algorithm consists of three key pillars (based on the well-established Wilson and Jungner classic screening principles [90]): condition, screening, and treatment, with each pillar comprising specific criteria and assigned weights [90]. Using available scientific evidence, the algorithm was used to assess inherited metabolic diseases, attributing scores to each category. Applying this evaluation method, MPS II attained a total score of 7 [48], which did not meet the recommended threshold for consideration in NBS programs across Europe (proposed cutoff score based on those conditions already screened for nationally in the UK: 8.5) [90].

Lin et al. (2022) examined the timely administration of ERT and/or HSCT to infants with a diagnosis of MPS II identified through NBS between August 2015 and April 2022 [53]. Patients were followed up for a maximum of 6 years. After ERT and/or HSCT administration, significant improvements in I2S activity, urinary DMB/Cre ratio, and DS and HS excretion were observed compared with baseline data. Timely referral of patients to medical specialists, early application of multidisciplinary care, and advancements in ERT and HSCT were suggested to have contributed to improved treatment outcomes [53].

Finally, a review by Ream et al., published in 2023, described a nationwide survey that was conducted to assess the readiness of NBS programs in the USA for implementing comprehensive screening for MPS II [29]. Among the 37 NBS programs analyzed across the USA, it was estimated that 8% of programs could implement MPS II NBS in less than 1 year, 24% in 1–2 years, 38% in 2–3 years, and that 30% would require more than 3 years. These projections did not consider potential state priorities that may take precedence over MPS II NBS implementation, such as incorporating other RUSP conditions or upgrading laboratory systems. Several barriers to MPS II screening were identified, including staffing challenges, competing NBS priorities, insufficient funding, and administrative approval obstacles.

The survey found that the additional cost of adding MPS II NBS per newborn child would range from USD 2 to USD 6, beyond regular NBS operating costs. Despite these challenges, NBS programs persist in expanding their screening panels. The survey revealed factors facilitating MPS II NBS implementation, such as the inclusion of the condition in the RUSP, increased funding and fees, ability to combine screening with other LSDs, advocacy efforts, and availability of a US Food and Drug Administration-approved laboratory kit [29].

## 4. Discussion

In total, this SLR identified 53 articles that referenced MPS II NBS, the vast majority of which were screening studies focusing on the validation of assays and/or reported results from NBS programs or pilots. As of July 2023, systematic NBS programs for MPS II were only found to exist in Taiwan, with state-wide introduction of MPS II NBS also identified in Illinois and Missouri. The first-stage screen in these existing NBS programs and in pilot initiatives typically involved quantification of I2S activity in DBS by MS/MS or fluorometry; positive screen cases were subsequently referred for further investigation and confirmation of a diagnosis, typically by evaluation of urine or DBS GAG concentrations and *IDS* sequencing. Some pilot and validation studies have also used assessment of plasma GAGs or urine or plasma GAG-derived disaccharides as an effective addition to confirm the diagnosis of MPS II. However, there is a paucity of evidence regarding the follow-up and outcomes of patients whose MPS II was identified via NBS screening.

NBS in Taiwan is thought to be one of the most comprehensive NBS systems in the world and has been consistently screening more than 95% of all newborns since 1993 [80]. NBS for MPS I, II, and VI in Taiwan commenced in 2015, with screening using a multiplex assay including MPS II initiating in 2018. Illinois became the first state in the USA to implement population-based NBS for MPS II at the end of 2017, followed by Missouri at the end of 2018. Pilot studies have been reported in at least three states in the USA [22,71,77]. Subsequent to the time frame of our SLR, a publication described an NBS pilot study for MPS II in North Carolina using a laboratory-developed LC–MS/MS test for I2S activity in DBS; results will be reported for all infants who screened positive for MPS II, including the relationship between I2S activity and GAG levels and the identified *IDS* genotypes [91]. An increase in the number of states adopting MPS II within their NBS programs is expected, given the 2022 recommendation to add MPS II to the NBS RUSP [28]. However, incorporation may be slow, given experiences with MPS I: although MPS I was added to the NBS RUSP in 2015 [92], it was reported that only 17 US states had included MPS I in their NBS panel as of May 2019 [93], with 43 states universally screening for MPS I in 2023 [94]. Improved collaboration among all relevant stakeholders involved in implementing screening programs may help to facilitate the establishment of a new program. In Iowa, the Congenital and Inherited Disorders Advisory Committee (CIDAC) is required by law to review conditions for potential addition to the state’s NBS panel in the 12 months from the inclusion of that condition in the RUSP. Moreover, after a recommendation from CIDAC to screen for the condition, the law requires the Iowa Newborn Screening Program (INSP) to implement screening for the condition in the subsequent 18 months. The Iowa Department of Health and Human Services worked with CIDAC leadership to establish a subcommittee for management of the Iowa NBS panel. MPS II was the first condition to be assessed by the subcommittee, and results from the review process highlighted that “screening for the condition has a high certainty of significant net benefits and screening has high or moderate feasibility” and that the “INSP has developmental readiness to screen within 18 months” [91]. According to the US Association of Public Health Laboratories, as of 15 April 2024, Illinois, Kentucky, Missouri, Pennsylvania, and West Virginia were the only US states to have implemented universal screening for MPS II despite its inclusion in the RUSP [94]. There was no evidence of other countries incorporating MPS II in their national NBS program in the literature. Across Europe, there are great disparities in screening programs. As mentioned, according to an NBS evaluation algorithm described by Burlina et al. in 2022 [90] and used by Jones and colleagues [48], MPS II did not meet the recommended threshold for consideration in NBS programs across Europe. However, the originally assigned scores, notably for the categories “Condition” and “Screening”, may warrant reassessment [48]. For example, in Jones et al., MPS II was assigned a score of 0 out of 0.5 points for “There is a rapidly progressing form” and of 0 out of 1 point for “The condition can be fatal by adolescence” [48]. In reality, however, MPS II can progress rapidly and be fatal by adolescence [2,95]. Moreover, as reported by NBS programs from Taiwan and the USA [23,27,34,36,37,38,39,41,66,67,73], the frequency of MPS II is greater than that acknowledged by the algorithm (where it was assigned 0.5 points based on a frequency of between 1 in 150,000 and 1 in 250,000). Finally, the algorithm did not recognize that a DBS test is available and has been used in some US states since 2017 [34,36,37,38]. Although the algorithm aims to provide an objective evaluation of the available evidence, the authors do acknowledge that it is not possible to eliminate the subjectivity of NBS completely [90]. Indeed, if the scores assigned to MPS II were to be adjusted based on these points, the disease would comfortably exceed the threshold for NBS.

One of the clinical benefits of NBS programs, when widely implemented across large populations, is an improved understanding of disease prevalence. It is often cited that MPS II occurs in approximately 1 in 100,000–170,000 male births [96]. In this SLR, the prevalence of MPS II as measured by NBS programs ranged from 1 in 24,581 to 1 in 60,671 births in Taiwan and from 1 in 73,290 to 1 in 105,214 births in the USA. The higher prevalence of MPS II in Taiwan and Japan compared with the USA may be explained by the higher allele frequency of *IDS* R468 variants in East Asia [97], which accounted for nearly half (42.9%) of all variants in a study with 14 Taiwanese patients with MPS II [98] and 11.6% of all variants in a group of 43 Japanese individuals with MPS II [99]. Given the screening rate in Taiwan and that MPS II screening has been carried out since 2015, these rates can likely be considered representative of the Taiwanese population. The MPS II birth prevalence rates from Illinois, Missouri, and Washington seem relatively high but have decreased with expanded screening and numbers of newborns screened. This may be considered representative of the US states in which the data were obtained but not necessarily of the US population in general. Indeed, the ability to identify regional differences in disease prevalence can lead to a better understanding of the disease and promote investigation into the underlying causes of any disparities.

The existing NBS programs use measurement of I2S activity via MS/MS or fluorometry. MS/MS permits rapid, sensitive, and accurate measurement of enzyme activity, with multiplexing facilitating screening for various LSDs from a single DBS specimen. Careful selection of thresholds for low I2S activity is required to reconcile sensitivity and specificity and to identify the optimal cutoffs to minimize false positives. Describing their early experience in 2015, Chan et al. described plans to lower cutoffs after the vast majority of newborns with enzymatic activities below the 30% cutoff obtained a false positive result [39]. More recently, NBS programs have defined a positive screen as I2S activity of 10% of normal or lower [22,36,37].

Fluorometric I2S assays are a common alternative to MS/MS and are becoming increasingly popular because they are less expensive, more widely available, and easier to use than mass spectrometry. The analytical ranges for MS/MS assays have been reported to be more than 50-fold higher than traditional fluorometric assays [50]. However, the application of digital microfluidic chip technology has increased throughput [100], making these assays a viable alternative to mass spectrometry for first-stage screening. MS/MS quantification of GAGs and GAG-derived disaccharides is another strategy for NBS and, although not necessarily an alternative to quantification of I2S activity, can be an effective complimentary approach, having been shown to reduce false positive and false negative rates to 0% [25,27].

GAG biomarker analysis is considered more powerful than genotyping as a second-tier testing for disease diagnosis and prognosis [101]. A study from 2022 showed that second-tier GAG biomarker analysis can dramatically reduce the false positive rate in NBS following measurement of enzymatic activity in DBS as the preferred first-tier method in NBS for mucopolysaccharidoses [102]. Two different GAG mass spectrometry methods were analyzed, the “classic” internal disaccharide biomarker analysis and the endogenous non-reducing end (NRE) biomarker method, which detects the NRE of the GAG polymer; findings from this research supported the use of the NRE biomarker method as a second-tier GAG analysis of newborn DBS to reduce the false positive rate [102].

Technologies regarding NBS in general are continuing to evolve in the search for cost-effective, efficient, specific, and sensitive solutions. For example, high-throughput MS/MS provides similar sensitivity and specificity to LC–MS/MS-based methods for the evaluation of HS [63]. A novel method that measures urine levels of low-molecular-weight GAG fragments (oligosaccharides) with non-reducing termini has been able to provide a diagnostic oligosaccharide signature unique for each subtype of MPS, without the need for prior deconstruction of the high-molecular-weight GAG, and showing 100% sensitivity and specificity [103]. The ability to assay both I2S activity and GAG levels from the same single DBS has also been demonstrated [104,105], and the optimal process may be to perform I2S activity and GAG quantification and genetic testing in multistage analysis from a single DBS.

Genotype analysis is often used to confirm a diagnosis of MPS II after a positive screening result, but it can be inconclusive because of the presence of variants with unknown pathogenic significance. In parallel with the ongoing emergence of novel variants, there have been significant research efforts leading to an improved understanding of the relationship between genotype and phenotype in MPS II [106,107,108]. However, the possible identification of presumed pseudodeficiency alleles and genomic VUS remains important to acknowledge in MPS II, and some patients with VUS without a clear MPS II diagnosis may need to undergo long-term monitoring for emergence of symptoms [34,39,54]. In the 2022 report on the Taiwanese NBS program by Lin et al., follow-up every 6 months was undertaken for 151 infants with suspected MPS II/I2S pseudodeficiency [53]. Genetic counseling and screening were also provided and were particularly important for maternal-side male senior relative screening for the 16 novel *IDS* variants identified in this study. This follow-up strategy needs to be further investigated, and international recommendations or consensus would be helpful.

The biggest shift in NBS in general is likely to come with broader implementation of next-generation sequencing panels, whole-exome sequencing (WES), and whole-genome sequencing (WGS) [44]. These technologies bring considerable debate regarding the benefits and potential harms for the individual throughout their lifetime, for parents and the wider family, for others in society, and for healthcare systems [109], which are beyond the scope of this review but also warrant mention, particularly given the VUS in MPS II. The risk–benefit of WES or WGS in population-based screening is being debated [109,110,111]. It is considered that the medical and research communities’ understanding of, and ability to interpret, genomic variants do not justify the use of WES or WGS in population-based NBS. Indeed, reporting of all VUS in *IDS* identified by WES or WGS would likely result in a high false positive rate and impose a substantial burden on healthcare systems to provide biochemical testing and long-term clinical follow-up of newborns with these variants. Building sustainable NBS programs that incorporate WES or WGS will require an ongoing assessment evaluating health outcomes, psychosocial outcomes, costs, cost-effectiveness, and long-term contact with families that have been screened. The ongoing Screen4Care project, using and validating different genome screening strategies, aims to assess scalability of genetic NBS for rare diseases deemed treatable or actionable to reduce the cost of screening per disease and per individual [111].

The creation of NBS programs is a major public health achievement that has decreased the morbidity and mortality of inborn errors of metabolism [112]. Preliminary evidence from Taiwan from patients with various types of MPS showed a reduction in the age of diagnosis of various types of MPS since the implementation of NBS and a positive correlation between age at diagnosis and age at death [113]. In the absence of specific information on MPS II, published surveys of the patient and family experience of NBS in other LSDs, including other forms of MPS and Fabry, Gaucher, Krabbe, and Pompe diseases, all report that the majority of respondents support the implementation of NBS [114,115,116,117,118,119,120,121,122]. A survey of adults with late-onset LSDs (Fabry disease, Gaucher disease, or late-onset Pompe disease) found that more than 90% of the 36 participants were in favor of NBS. For families, the benefits of NBS included the potential to avoid diagnostic delays and to initiate treatment earlier and the ability to prevent irreversible organ damage. The results of NBS may also inform life decisions such as relationships, career, and lifestyle choices [119]. The ability for parents to make future reproductive decisions based on their child’s NBS result was also considered a benefit by some participants. Psychological burdens were obviously mentioned, as were concerns about medical insurance discrimination. In agreement with other studies, our SLR identifies the need for similar research to be conducted in families with MPS II and also for a better understanding of the impact of diagnosis after NBS and the experience of clinical follow-up for patients with MPS II [47,54]. An important broader benefit of NBS for rare diseases such as MPS II is the potential increase in genomic and phenotypic information, which would further enhance our understanding of the disease.

The importance of patient advocacy groups in supporting families affected by rare genetic disorders should not be underestimated [123]. In a survey of parents of children with rare diseases, information on patient support groups was one of the top five topics searched on the internet at the time of diagnosis [124]. Parent support groups for patients affected by LSDs contributed to the movement for more research fundraising and advocated for policies to include MPS II in routine NBS [47]. The successful application for MPS II to be added to the RUSP was submitted and defended by the US National MPS Society, with patient testimonies playing a key role [28,85].

It has been suggested that families of patients with MPS II should be offered genetic counseling and support services [47,54]. The lack of resources available for patient follow-up was voiced as a frustration by some US HCPs in the survey by Lisi et al. [54] but appeared to vary from state to state. Overall, it is clear that more research is essential to identify the holistic impact of MPS II NBS on families after receiving a diagnosis.

### Strengths and Limitations

This was a comprehensive SLR capturing relevant literature on NBS programs for MPS II, designed and conducted using robust methodology in accordance with the 2020 PRISMA guidelines [31]. A quality assessment of the literature was not performed because no appropriate risk-of-bias tools could be identified to assess screening studies (the predominant type of article identified) adequately. Owing to the limited number of articles reporting views on MPS II NBS from HCPs or families of patients, insights may not reflect all opinions or experiences.

## 5. Conclusions

The current state of NBS for MPS II has been described in this study. Systematic NBS programs for MPS II are currently only carried out in Taiwan and parts of the USA. However, following the addition of MPS II to the RUSP, NBS programs for MPS II should become more widespread in US states; as of April 2024, only five states have implemented the recommendation, but it has been suggested that approximately a quarter of states might be expected do so in the next 2 years. There is a notable evidence gap concerning the follow-up and treatment of patients whose MPS II was diagnosed through NBS. Clinical follow-up studies and surveys of the patient experience are required to understand the full impact of NBS on patients with MPS II and their families.

## Figures and Tables

**Table 1:** Status of MPS II NBS programs and pilots.

Region	NBS Program Details	Program Initiation
Existing NBS programs		
Taiwan [23,27,39,41,53,66,67]	The Taiwanese NBS program was established in 2015 and screens approximately 35% of newborns per year. From August 2015 to April 2022, 546,040 infants were screened for MPS II using MS/MS for I2S enzyme activity in DBS. For newborns with consistent below-cutoff results, genotyping was performed on DNA extracted from DBS. In total, 223 positive screens were referred and nine cases were subsequently confirmed. Samples with I2S activity below the predefined threshold (6.5 μmol/L/h) were retested in duplicate. Samples with I2S activity below a lower threshold of 2.2 μmol/L/h were designated as positive screens and referred for MPS confirmation at the Mackay Memorial Hospital.	2015
USA, Illinois [36,37,38,73]	Illinois became the first state in the USA to implement population-based NBS for MPS II in December 2017. As of April 2022, 700,616 samples from 586,323 infants had been tested, leading to eight confirmed diagnoses. A positive screen for MPS II was defined as I2S activity ≤10% of the daily median activity. Samples with I2S activity >10–13% of the daily median activity were classified as borderline, and a second specimen was requested. Infants with a positive result were referred for diagnostic testing, which was performed at commercial laboratories determined by the consultant evaluating the patient.	2017
USA, Missouri [34]	In Missouri, prospective NBS for MPS II began in November 2018 using a fluorometric enzyme assay to measure I2S activity in DBS samples. By June 2020, 146,954 samples had been screened, and two newborns had received a diagnosis of severe MPS II after positive first- and second-tier screens and positive confirmatory test results. Samples with I2S activity below the “retest cutoff” (initially 40 μmol/L/h; adjusted to 25 μmol/L/h from 2 August 2019) were repeated in duplicate; of these, samples with average activity below the “high-risk cutoff” (initially 35 μmol/L/h; adjusted to 20 μmol/L/h from 2 August 2019) were sent for second-tier testing. From November 2018 to December 2019, second-tier screening was performed via molecular sequencing at Greenwood Genetic Center; from January to June 2020, second-tier screening was performed by evaluation of DBS GAG concentrations at Mayo Clinic Laboratories. For samples with a positive second-tier result, the newborn was referred to a contracted follow-up center for confirmatory diagnostic testing.	2018
Pilot NBS programs		
Japan, Tokyo [79]	As a result of more treatments becoming available in Japan, NBS for treatable LSDs is being piloted in Tokyo metropolitan and surrounding areas using LC–MS/MS as the screening method for detecting LSDs.	NR
USA, New York [77]	ScreenPlus is a comprehensive NBS program expected to enroll approximately 150,000–175,000 infants born at eight hospitals in New York over a 5-year period. Using a megaplex LC–MS/MS screening platform for first-tier testing, ScreenPlus will also include second-tier biomarkers and third-tier DNA testing to enhance the accuracy of screening for a range of disorders, including MPS II.	2020
USA, Washington [22]	A pilot program was initiated in Washington in 2019 using a two-tier strategy comprising LC–MS/MS and subsequent genotype sequencing on anonymized DBS.	NR

DBS, dried blood spot(s); I2S, iduronate-2-sulfatase; LC, liquid chromatography; LSD, lysosomal storage disease; MPS, mucopolysaccharidosis; MS/MS, tandem mass spectrometry; NBS, newborn screening; NR, not reported.

**Table 3:** MPS II NBS technologies.

Region, Year	Technology
Argentina, Spain, 2010 [33]	Urine GAG quantification by multiplex DMB assay
USA, 2020 [34]	First-tier screening: DBS I2S activity by fluorometric assay Second-tier screening: gene sequencing/GAG analysis of DBS
USA, 2019 [36], 2020 [37], and 2023 [38]	DBS I2S activity by MS/MS Total GAG analysis using DMB incorporation and spectrophotometry GAG-derived disaccharide (HS and DS) quantification by ESI–MS/MS Molecular testing
Taiwan, 2019 [39]	DBS I2S activity by multiplex MS/MS Gene sequencing
USA, 2014 [40]	DBS I2S activity by multiplex LC–MS/MS
Taiwan, 2020 [23] and 2020 [66]	DBS I2S activity by multiplex LC–MS/MS Gene sequencing
Taiwan, 2019 [67]	DBS I2S activity by MS/MS Gene sequencing
Taiwan, 2021 [27]	DBS I2S activity by MS/MS
Taiwan, 2022 [41]	DBS I2S activity by MS/MS
Brazil*,* 2006 [42]	DBS I2S activity by fluorometric assay
Netherlands*,* 2012 [43]	DBS GAG-derived disaccharide quantification by LC–MS/MS
Morocco, ^a^ 2020 [45]	Urine GAG quantification by DMB assay Urine GAG quantification by semi-quantitative colorimetric assay Urine GAG quantification by thin-layer chromatography Urine I2S activity by fluorometric assay
USA, 2017 [68]	DBS I2S activity by multiplex MS/MS
Italy, Taiwan, USA*,* 2019 [46]	DBS I2S activity by multiplex MS/MS DBS I2S activity by multiplex DMF
USA, 2020 [69]	DBS I2S activity by multiplex LC–MS/MS
USA, 2020 [70]	Quantification of GAG-derived biomarkers by LC–MS/MS of DBS (proposed as a second-tier strategy)
USA, 2020 [49]	DBS/fibroblast lysate I2S activity by multiplex LC–MS/MS
USA, 2015 [50]	DBS I2S activity by multiplex MS/MS DBS I2S activity by multiplex fluorometric assay
USA, 2019 [51]	DBS I2S activity by multiplex MS/MS
South Korea*,* 2015 [52]	DBS I2S activity by multiplex LC–MS/MS
Taiwan, 2022 [53]	DBS I2S activity by MS/MS
USA, 2017 [55]	DBS I2S activity by multiplex LC–MS/MS
Italy, 2018 [56]	DBS GAG quantification by capillary electrophoresis using laser-induced fluorescence separation
Australia, 2004 [57]	DBS protein biomarker (LAMP1 and saposin C) quantification by time-resolved fluorescence dual assay and metabolite markers by LC–MS/MS
Australia, 2006 [58]	DBS I2S, LAMP1 and saposin C quantification by multiplex immunoassay
Canada, 2019 [59]	Urine GAG (DS and HS) quantification by ultra-performance LC–MS/MS
USA, 2022 [30]	First-tier screening: DBS I2S activity by MS/MS or microfluorometry Second-tier screening: molecular and/or biochemical testing
Japan, 2020 [60]	DBS I2S activity by multiplex LC–MS/MS assay
Japan, 2022 [79]	Quantification of lysosomal enzyme activity in urine samples by LC–MS/MS
USA, 2020 [72]	First-tier screening: plasma I2S activity by multiplex MS/MS Second-tier screening: urine GAGs and/or molecular analysis (method not defined)
USA, 2021 [73]	DBS I2S activity by multiplex MS/MS Urine GAG quantification (method not defined) Gene sequencing
Netherlands, 2014 [61]	DBS I2S activity by fluorometric assay
Morocco*,* 2020 [62]	Urine GAG (CS) quantification by DMB assay
USA, 2020 [22]	First-tier screening: DBS I2S activity by multiplex LC–MS/MS Second-tier screening: gene sequencing
NR*,* 2014 [63]	DBS GAG (HS) quantification by multiplex high-throughput MS/MS DBS GAG (HS) quantification by multiplex LC–MS/MS
Japan*,* 2020 [25]	DBS GAG (DS and HS) quantification by LC–MS/MS DBS I2S activity by fluorometric assay
NR, 2019 [74]	DBS GAG (not defined) quantification by LC–MS/MS
USA, 2017 [75]	DBS I2S assay by multiplex digital microfluidics
NR, 2013 [76]	DBS GAG (DS and HS) and disaccharide (not defined) quantification by LC–MS/MS
USA, 2007 [64]	DBS I2S activity by MS/MS
USA, 2020 [77]	First-tier screening: I2S activity by LC–MS/MS (sample type NR) Second-tier screening: biomarker quantification (method not defined) Third-tier screening: gene sequencing

^a^ Targeted screening program of patients with suspected MPS aged 9 months–15.6 years. CS, chondroitin sulfate; DBS, dried blood spot(s); DMB, dimethylmethylene blue; DS, dermatan sulfate; ESI, electrospray isotope dilution; GAG, glycosaminoglycan; HS, heparan sulfate; I2S, iduronate-2-sulfatase; KS, keratan sulfate; LAMP1, lysosomal-associated membrane protein 1; LC, liquid chromatography; MPS, mucopolysaccharidosis; MS/MS, tandem mass spectrometry; NBS, newborn screening; NR, not reported.

## Data Availability

The data included in this report are from the published literature; all articles meeting the search criteria are listed and full publication details are provided.

## Supplementary Materials

The following supporting information can be downloaded at: https://www.mdpi.com/article/10.3390/ijns10040071/s1, Table S1: Search strategy to identify studies for the SLR. Table S2: Eligibility criteria for studies identified by the SLR. Table S3: Summary of included articles on MPS II. Figure S1: PRISMA diagram of studies excluded from and included in the SLR.

## Abbreviations

CIDAC	Congenital and Inherited Disorders Advisory Committee
CS	chondroitin sulfate
Cre	creatinine
DBS	dried blood spot(s)
DMB	dimethylmethylene blue
DS	dermatan sulfate
ESI	electrospray isotope dilution
ERT	enzyme replacement therapy
GAG	glycosaminoglycan
HCP	healthcare professional
HS	heparan sulfate
HS-0S	heparan ΔDi-0S [2-acetamido-2-deoxy-4-O-(4-deoxy-α-L-*threo*-hex-4-enopyranosyluronic acid)-D-glucose]
HS-NS	heparan ΔDi-NS [2-deoxy-2-sulfamino 4-O-(4-deoxy-α-L-*threo*-hex-4-enopyranosyluronic acid)-D-glucose]
HSCT	hematopoietic stem cell transplantation
*IDS*	iduronate-2-sulfatase gene
I2S	iduronate-2-sulfatase
INSP	Iowa Newborn Screening Program
KS	keratan sulfate
LAMP1	lysosomal-associated membrane protein 1
LC	liquid chromatography
LSD	lysosomal storage disease
MPS	mucopolysaccharidosis
MS/MS	tandem mass spectrometry
NA	not applicable
NBS	newborn screening
NR	not reported
PRISMA	Preferred Reporting Items for Systematic Reviews and Meta-Analyses
RUSP	Recommended Uniform Screening Panel
SLR	systematic literature review
VUS	variant(s) of unknown significance
WES	whole-exome sequencing
WGS	whole-genome sequencing
