# Supplementary material for: A Systematic Literature Review on the Global Status of Newborn Screening for Mucopolysaccharidosis II
Source: Int J Neonatal Screen. 2024 Oct 10;10(4):71. doi: 10.3390/ijns10040071 (PMC11503380; doi:10.3390/ijns10040071)
Supplement: Supplementary file 1 [file IJNS-10-00071-s001.zip › IJNS-3139514-supplementary.pdf]

Supplementary Material

Supplementary Table S1. Search strategy to identify studies for the SLR.

Embase

#	Searches	Strategy
1	exp newborn screening/ or ((new born or new-born or newborn) adj3 screening).mp.	NBS
2	(mucopolysaccharidosis or (hunter* adj (syndrome or disease)) or MPS).mp.	MPS
3	exp lysosome storage disease/ or lysosomal storage disease?.mp.	LSD
4	2 or 3	MPS or LSD
5	1 and 4	

MEDLINE

#	Searches	Strategy
1	exp newborn screening/ or ((new born or new-born or newborn) adj3 screening).mp.	NBS
2	(mucopolysaccharidosis or (hunter* adj (syndrome or disease)) or MPS).mp.	MPS
3	exp lysosome storage disease/ or lysosomal storage disease?.mp.	LSD
4	2 or 3	MPS or LSD
5	1 and 4	

Cochrane libraries

#	Searches	Strategy
1	exp newborn screening/ or ((new born or new-born or newborn) adj3 screening).mp.	NBS
2	(mucopolysaccharidosis or (hunter* adj (syndrome or disease)) or MPS).mp.	MPS
3	exp lysosome storage disease/ or lysosomal storage disease?.mp.	LSD
4	2 or 3	MPS or LSD
5	1 and 4	

LSD, lysosomal storage disease; MPS, mucopolysaccharidosis; NBS, newborn screening.

Supplementary Table S2. Eligibility criteria for studies identified by the SLR.

**Category**	**Inclusion criteria**
Population	Patients undergoing NBS for any LSD (initial search) or MPS II (updated search)
Interventions	Not restricted by intervention
Comparator	Not restricted by comparators
Outcomes	Status<br>Countries with existing programs, pilot studies, or plans for NBS for any type of LSD (initial search) or MPS II (updated search)<br>Guidelines and recommendations for existing NBS programs (updated search only)<br>Technologies<br>Methodology/technologies in use for NBS (brief overview only)<br>Diagnostic testing validity and reliability assessments (initial search only)<br>Management of false positives and pseudodeficiency; use of additional testing to minimize false positives (updated search only)<br>Implementation<br>Barriers to implementation of NBS in different countries/regions (e.g., infrastructure, perceived cost–benefit ratio, meeting criteria to be added to screening panel)<br>Outcomes<br>Outcomes of NBS pilot studies/existing programs on mortality and morbidity, including estimated incidence, prevalence,[a] and follow-up experience; impact on mortality and morbidity outcomes<br>Patient follow-up and management (referral and support systems, uncertainty around prognosis/genotype–phenotype relationships, treatment decisions)
Study designs	Not restricted by study design
Date restrictions	Not restricted by date
Country restrictions	Not restricted by country
Language	English (initial search) or not limited by publication language (updated search)

[a] Prevalence estimates extracted if presented in included studies.
LSD, lysosomal storage disease; MPS, mucopolysaccharidosis; NBS, newborn screening; SLR, systematic literature review.

Supplementary Table S3. Summary of included articles on MPS II.

First author, year (publication type)	Study design	Country	Number of patients/samples	LSDs covered	Status	Technology	Estimated birth prevalence	Follow-up and outcomes
**Total number of articles on topic**					21	44	17	6
**Burton, 2023 [38] (full publication)**	Laboratory study	USA	586,323 infants screened	MPS II	✓	✓	✓	
**Ream, 2023 [29] (full publication)**	Narrative review	USA	NR	MPS II	✓			✓
**Brower, 2022 [35] (full publication)**	Narrative review and survey	USA	36 experts completed the survey	Various LSDs, including MPS II	✓			
**Chuang, 2022 [41] (full publication)**	Laboratory study	Taiwan	546,040 infants screened for MPS II	MPS I, II, IVA, and VI	✓	✓	✓	
**Jones, 2022 [48] (full publication)**	Evaluative analysis	Europe	NR	Various IMDs, including MPS II				✓
**Lin, 2022 [53] (full publication)**	Prospective observational study	Taiwan	548,624 newborns screened (study is focused on the 202 infants referred for confirmatory diagnosis)	MPS II	✓	✓	✓	✓
**Millington, 2022 [30] (full publication)**	Editorial	USA	NR	MPS II	✓	✓	✓	
**Okuyama, 2022 [79] (abstract)**	Laboratory study	Japan	Over 30,000 newborns	MPS I, II, and VI, and Pompe disease	✓	✓	✓	
**Pique, 2022 [78] (abstract)**	Cross-sectional study	USA	NR	Various LSDs, including MPS II	✓			
**Chuang, 2021 [27] (full publication)**	Laboratory study	Taiwan	More than 600,000 infants total, including 307,731 screened for MPS II	MPS I, II, VI, and IVA	✓	✓	✓	
**Quadri, 2021 [73] (abstract)**	Laboratory study	USA	24 newborns	MPS II	✓	✓		
**Bilyeu, 2020 [34] (full publication)**	Laboratory study	USA	NR	MPS II	✓	✓	✓	
**Burton, 2020 [37] (full publication)**	Laboratory study	USA	339,269 infants screened	MPS II	✓	✓	✓	
**Chien, 2020 [66] (abstract)**	Screening study	Taiwan	73,743 newborns screened	Fabry disease, Gaucher disease, MPS I, II, IIIB, IVA, and VI, and Pompe disease	✓	✓	✓	

First author, year (publication type)	Study design	Country	Number of patients/samples	LSDs covered	Status	Technology	Estimated birth prevalence	Follow-up and outcomes
**Chien, 2020 [23] (full publication)**	Laboratory study	Taiwan	73,743 newborns screened	Fabry disease, Gaucher disease, MPS I, II, IIIB, IVA, and VI, and Pompe disease	✓	✓	✓	
**Fdil, 2020 [45] (full publication)**	Laboratory study[a]	Morocco	88 patients with suspected MPS	MPS I and II		✓		
**Gelb, 2020 [69] (abstract)**	Laboratory study	USA	NR	Fabry disease, Gaucher disease, Krabbe disease, MPS I, II, IIIA, IIIB, IIIC, IIID, IVA, VI, and VII, Niemann–Pick disease type A/B/C, Pompe disease, and others		✓		
**Gelb, 2020 [70] (abstract)**	Laboratory study	USA	NR	MPS I, II, IIIA, IIIB, IVA, IV, and VII		✓		
**Khaledi, 2020 [49] (full publication)**	Laboratory study	USA	NR	All MPS types except MPS IX		✓		
**Oguni, 2020 [60] (full publication)**	Laboratory study	Japan	672 DBS samples from healthy newborns and 23 patients with MPS	MPS I, II, IIIB, IVA, and VI		✓		
**Pollard, 2020 [72] (abstract)**	Laboratory study	USA	446 infants	MPS I and II	✓	✓		
**Sabir, 2020 [62] (full publication)**	Laboratory study	Morocco	47 patients with MPS	MPS I and II		✓		
**Scott, 2020 [22] (full publication)**	Laboratory study	USA	~106,000 newborns	MPS II, IIIB, IVA, VI, and VII	✓	✓	✓	
**Stapleton, 2020 [25] (full publication)**	Laboratory study	Japan	18,222 DBS samples	MPS II		✓	✓	
**Wasserstein, 2020 [77] (abstract)**	Laboratory study	USA	150,000–175,000 infants	CLN2, CTX, Fabry disease, Gaucher disease, lysosomal acid lipase deficiency, MLD, MPS II, IIIB, IVA, VI, and VII, and Niemann–Pick	✓	✓		

First author, year (publication type)	Study design	Country	Number of patients/samples	LSDs covered	Status	Technology	Estimated birth prevalence	Follow-up and outcomes
				disease type A/B/C				
**Burton, 2019 [36] (full publication)**	Laboratory study	USA	162,000 infant samples screened	MPS II	✓	✓	✓	
**Chan, 2019 [39] (full publication)**	Laboratory study	Taiwan	130,237 newborns screened	MPS I, II, and VI	✓	✓	✓	
**Chuang, 2019 [67] (abstract)**	Laboratory study	Taiwan	280,984 infants screened for MPS	MPS I, II, and VI	✓	✓	✓	
**Fabie, 2019 [44] (full publication)**	Narrative review	USA	NR	Fabry disease, Gaucher disease, Krabbe disease, MPS I and II, Niemann–Pick disease type A/B, and Pompe disease	✓			
**Gelb, 2019 [46] (full publication)**	SLR	Italy, Taiwan, USA	NR	Fabry disease, Gaucher disease, Krabbe disease, MLD, MPS I, II, IIIA, IIIB, IVA, VI, and VII, Niemann–Pick disease type A/B/C, Pompe disease, and others		✓		
**Kumar, 2019 [51] (full publication)**	Laboratory study	USA	NR	Fabry disease, Gaucher disease, Krabbe disease, MPS I, II, IIIB, IVA, VI, and VII, Niemann–Pick disease type A/B, and Pompe disease		✓		
**Menkovic, 2019 [59] (full publication)**	Laboratory study	Canada	500 urine samples	MPS I, II, III, VI, and VII		✓		
**Tomatsu, 2019 [74] (abstract)**	Laboratory study	NR	17,467 DBS samples from newborns and 14 DBS samples from newborns with MPS (two with MPS II)	MPS I, II, and III		✓		

First author, year (publication type)	Study design	Country	Number of patients/samples	LSDs covered	Status	Technology	Estimated birth prevalence	Follow-up and outcomes
**Joseph, 2018 [47] (full publication)**	SLR	NR	NR	MPS II			✓	✓
**Maccari, 2018 [56] (abstract)**	Laboratory study	Italy	600 healthy newborns and six newborns with MPS (MPS II, n = 2)	MPS unspecified		✓		
**Gelb, 2017 [68] (abstract)**	Laboratory study	USA	~40,000 newborns screened	CLN1, CLN2, CTX, Krabbe disease, MLD, MPS I, II, IIIB, IVA, VI, and VII, Niemann–Pick disease type C, and Pompe disease		✓		
**Liu, 2017 [55] (full publication)**	Laboratory study	USA	DBS samples from 62 healthy newborns	CLN2 and MPS I, II, IIIB, IVA, VI, and VII		✓		
**Ullal, 2017 [75] (abstract)**	Laboratory study	USA	200 presumed normal and 26 MPS II DBS samples	MPS I and II, and Pompe disease		✓		
**Lisi, 2016 [54] (full publication)**	Survey	USA, Canada, India	38 HCPs	Fabry disease, Gaucher disease, Krabbe disease, MPS I and II, and Pompe disease				✓
**Kumar, 2015 [50] (full publication)**	Laboratory study	USA	NR	MPS II, IVA, and VI		✓		
**Lee, 2015 [52] (article)**	Laboratory study	South Korea	DBS from 110 healthy newborns and from three patients with MPS II	MPS II		✓		
**Chennamaneni, 2014 [40] (full publication)**	Laboratory study	USA	NR	MPS I, II, and VI		✓		
**Matern, 2014 [71] (abstract)**	Laboratory study	USA	100,000 newborns	Various LSDs, including MPS II			✓	
**Ruijter, 2014 [61] (full publication)**	Laboratory study	Netherlands	1426 newborns	MPS II		✓		
**Shimada, 2014 [63] (full publication)**	Laboratory study	NR	Blood (plasma or serum) samples from 55 patients	MPS II, III, and IV		✓		

First author, year (publication type)	Study design	Country	Number of patients/samples	LSDs covered	Status	Technology	Estimated birth prevalence	Follow-up and outcomes
			with MPS II; DBS sample from one patient with MPS II					
**van Vlies, 2013 [76] (abstract)**	Laboratory study	NR	One patient	MPS I, II, and III		✓		
**de Ruijter, 2012 [43] (full publication)**	Laboratory study	Netherlands	18 newborn DBS samples screened	MPS I, II, and III		✓		
**Alonso-Fernández, 2010 [33] (full publication)**	Laboratory study	Argentina/Spain	903 newborn samples screened	MPS I, II, and VI		✓		
**Yamaguchi, 2008 [65] (full publication)**	Narrative review	Japan	NR	Various LSDs, including MPS II				✓
**Wang, 2007 [64] (full publication)**	Laboratory study	USA	13 patients with MPS II and 57 randomly chosen newborns	MPS II		✓		
**Civallero, 2006 [42] (full publication)**	Laboratory study	Brazil	NR	Fabry disease, Gaucher disease, MPS I, II, IIIB, VI, and VII, and others		✓		
**Meikle, 2006 [58] (full publication)**	Laboratory study	Australia, Denmark	Samples from 92 individuals after diagnosis of an LSD	MLD, MPS I, II, IIIA, and VI, and Niemann–Pick disease type A/B		✓		
**Meikle, 2004 [57] (full publication)**	Laboratory study	Denmark, Australia	Guthrie cards retrieved from 47 patients with LSDs (n = 47)	Fabry disease, Gaucher disease, I-cell disease, Krabbe disease, MPS II, IVA, and IIIA, Pompe disease, and Tay–Sachs disease		✓		

[a] Targeted screening program of patients with suspected MPS aged 9 months–15.6 years.
CLN, neuronal ceroid lipofuscinosis; CTX, cerebrotendinous xanthomatosis; DBS, dried blood spot(s); IMD, inherited metabolic disease; HCP, healthcare professional; LSD, lysosomal storage disease; MLD, metachromatic leukodystrophy; MPS, mucopolysaccharidosis; NBS, newborn screening; NR, not reported; SLR, systematic literature review.

Supplementary Figure 1. PRISMA diagram of studies excluded from and included in the SLR. MPS, mucopolysaccharidosis; PRISMA, Preferred Reporting Items for Systematic reviews and Meta-Analyses; SLR, systematic literature review.

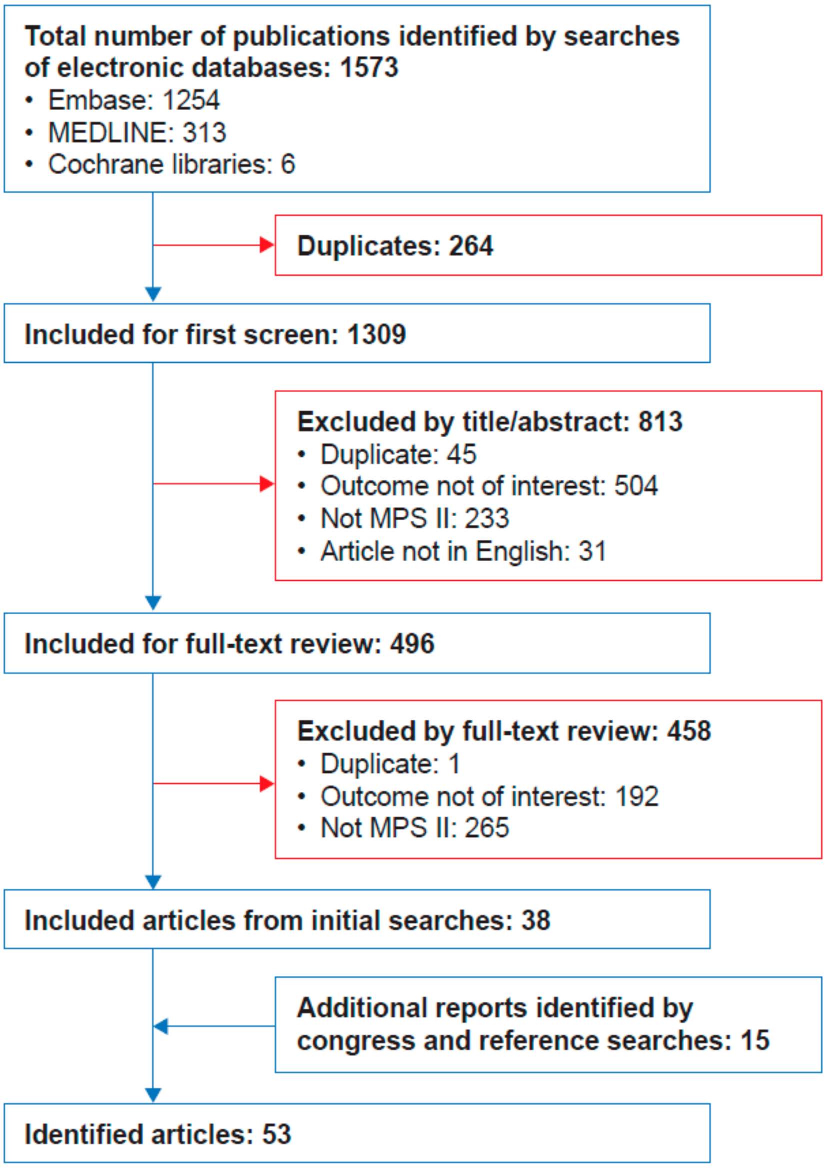

References

1. Gelb, M.H.; Scott, C.R.; Turecek, F. Newborn screening for lysosomal storage diseases. *Clinical Chemistry* **2015**, *61*, 335-346, doi:http://dx.doi.org/10.1373/clinchem.2014.225771.
2. Neufeld, E.; Muenzer, J. The mucopolysaccharidoses. In *The metabolic and molecular bases of inherited disease*, Scriver, C., Beaudet, A., Sly, W., Valle, D., Eds.; McGraw-Hill: New York, 2001; pp. 3421-3452.
3. Lau, H.; Harmatz, P.; Botha, J.; Audi, J.; Link, B. Clinical characteristics and somatic burden of patients with mucopolysaccharidosis II with or without neurological involvement: An analysis from the Hunter Outcome Survey. *Mol Genet Metab Rep* **2023**, *37*, 101005, doi:https://dx.doi.org/10.1016/j.ymgmr.2023.101005.
4. Baehner, F.; Schmiedeskamp, C.; Krummenauer, F.; Miebach, E.; Bajbouj, M.; Whybra, C.; Kohlschutter, A.; Kampmann, C.; Beck, M. Cumulative incidence rates of the mucopolysaccharidoses in Germany. *J Inherit Metab Dis* **2005**, *28*, 1011-1017, doi:https://dx.doi.org/10.1007/s10545-005-0112-z.
5. Nelson, J.; Crowhurst, J.; Carey, B.; Greed, L. Incidence of the mucopolysaccharidoses in Western Australia. *Am J Med Genet A* **2003**, *123A*, 310-313, doi:https://dx.doi.org/10.1002/ajmg.a.20314.
6. Ferreira, C.R.; Gahl, W.A. Lysosomal storage diseases. *Transl Sci Rare Dis* **2017**, *2*, 1-71, doi:https://dx.doi.org/10.3233/TRD-160005.
7. Burton, B.K.; Giugliani, R. Diagnosing Hunter syndrome in pediatric practice: practical considerations and common pitfalls. *Eur J Pediatr* **2012**, *171*, 631-639, doi:https://dx.doi.org/10.1007/s00431-012-1703-y.
8. Wraith, J.E.; Scarpa, M.; Beck, M.; Bodamer, O.A.; De Meirleir, L.; Guffon, N.; Meldgaard Lund, A.; Malm, G.; Van der Ploeg, A.T.; Zeman, J. Mucopolysaccharidosis type II (Hunter syndrome): a clinical review and recommendations for treatment in the era of enzyme replacement therapy. *Eur J Pediatr* **2008**, *167*, 267-277, doi:https://dx.doi.org/10.1007/s00431-007-0635-4.
9. Scarpa, M.; Almássy, Z.; Beck, M.; Bodamer, O.; Bruce, I.A.; De Meirleir, L.; Guffon, N.; Guillen-Navarro, E.; Hensman, P.; Jones, S.; et al. Mucopolysaccharidosis type II: European recommendations for the diagnosis and multidisciplinary management of a rare disease. *Orphanet J Rare Dis* **2011**, *6*, 72, doi:http://dx.doi.org/10.1186/1750-1172-6-72.
10. Bunge, S.; Rathmann, M.; Steglich, C.; Bondeson, M.-L.; Tylki-Szymanska, A.; Popowska, E.; Gal, A. Homologous nonallelic recombinations between the iduronate-sulfatase gene and pseudogene cause various intragenic deletions and inversions in patients with mucopolysaccharidosis type II. *European Journal of Human Genetics* **1998**, *6*, 492-500, doi:https://dx.doi.org/10.1038/sj.ejhg.5200213.
11. D'Avanzo, F.; Rigon, L.; Zanetti, A.; Tomanin, R. Mucopolysaccharidosis type II: one hundred years of research, diagnosis, and treatment. *Int J Mol Sci* **2020**, *21*, doi:https://dx.doi.org/10.3390/ijms21041258.
12. Muenzer, J.; Wraith, J.E.; Beck, M.; Giugliani, R.; Harmatz, P.; Eng, C.M.; Vellodi, A.; Martin, R.; Ramaswami, U.; Gucsavas-Calikoglu, M.; et al. A phase II/III clinical study of enzyme replacement therapy with idursulfase in mucopolysaccharidosis II (Hunter syndrome). *Genet Med* **2006**, *8*, 465-473, doi:https://dx.doi.org/10.1097/01.gim.0000232477.37660.fb.
13. Whiteman, D.A.; Kimura, A. Development of idursulfase therapy for mucopolysaccharidosis type II (Hunter syndrome): the past, the present and the future. *Drug Des Devel Ther* **2017**, *11*, 2467-2480, doi:https://dx.doi.org/10.2147/DDDT.S139601.
14. Muenzer, J.; Jones, S.A.; Tylki-Szymanska, A.; Harmatz, P.; Mendelsohn, N.J.; Guffon, N.; Giugliani, R.; Burton, B.K.; Scarpa, M.; Beck, M.; et al. Ten years of the Hunter Outcome Survey (HOS): insights, achievements, and lessons learned from a global patient registry. *Orphanet J Rare Dis* **2017**, *12*, 82, doi:https://dx.doi.org/10.1186/s13023-017-0635-z.
15. Muenzer, J.; Beck, M.; Eng, C.M.; Giugliani, R.; Harmatz, P.; Martin, R.; Ramaswami, U.; Vellodi, A.; Wraith, J.E.; Cleary, M.; et al. Long-term, open-labeled extension study of idursulfase in the treatment of Hunter syndrome. *Genet Med* **2011**, *13*, 95-101, doi:https://dx.doi.org/10.1097/GIM.0b013e3181fea459.

16. Muenzer, J.; Giugliani, R.; Scarpa, M.; Tylki-Szymanska, A.; Jego, V.; Beck, M. Clinical outcomes in idursulfase-treated patients with mucopolysaccharidosis type II: 3-year data from the hunter outcome survey (HOS). *Orphanet J Rare Dis* **2017**, *12*, 161, doi:https://dx.doi.org/10.1186/s13023-017-0712-3.
17. Burton, B.K.; Jego, V.; Mikl, J.; Jones, S.A. Survival in idursulfase-treated and untreated patients with mucopolysaccharidosis type II: data from the Hunter Outcome Survey (HOS). *J Inherit Metab Dis* **2017**, *40*, 867-874, doi:http://dx.doi.org/10.1007/s10545-017-0075-x.
18. McBride, K.L.; Berry, S.A.; Braverman, N.; Committee, A.T. Treatment of mucopolysaccharidosis type II (Hunter syndrome): a Delphi derived practice resource of the American College of Medical Genetics and Genomics (ACMG). *Genet Med* **2020**, *22*, 1735-1742, doi:https://dx.doi.org/10.1038/s41436-020-0909-z.
19. Schulze-Frenking, G.; Jones, S.A.; Roberts, J.; Beck, M.; Wraith, J.E. Effects of enzyme replacement therapy on growth in patients with mucopolysaccharidosis type II. *J Inherit Metab Dis* **2011**, *34*, 203-208, doi:https://dx.doi.org/10.1007/s10545-010-9215-2.
20. Muenzer, J. Overview of the mucopolysaccharidoses. *Rheumatology* **2011**, *50*, v4-v12, doi:https://dx.doi.org/10.1093/rheumatology/ker394.
21. Camargo Neto, E.; Schulte, J.; Pereira, J.; Bravo, H.; Sampaio-Filho, C.; Giugliani, R. Neonatal screening for four lysosomal storage diseases with a digital microfluidics platform: initial results in Brazil. *Genetics and Molecular Biology* **2018**, *41*, 414-416, doi:http://dx.doi.org/10.1590/1678-4685-gmb-2017-0227.
22. Scott, C.R.; Elliott, S.; Hong, X.; Huang, J.Y.; Kumar, A.B.; Yi, F.; Pendem, N.; Chennamaneni, N.K.; Gelb, M.H. Newborn screening for mucopolysaccharidoses: results of a pilot study with 100 000 dried blood spots. *Journal of Pediatrics* **2020**, *216*, 204-207, doi:http://dx.doi.org/10.1016/j.jpeds.2019.09.036.
23. Chien, Y.H.; Lee, N.C.; Chen, P.W.; Yeh, H.Y.; Gelb, M.H.; Chiu, P.C.; Chu, S.Y.; Lee, C.H.; Lee, A.R.; Hwu, W.L. Newborn screening for Morquio disease and other lysosomal storage diseases: results from the 8-plex assay for 70,000 newborns. *Orphanet Journal of Rare Diseases* **2020**, *15*, 38, doi:http://dx.doi.org/10.1186/s13023-020-1322-z.
24. Liao, H.C.; Chiang, C.C.; Niu, D.M.; Wang, C.H.; Kao, S.M.; Tsai, F.J.; Huang, Y.H.; Liu, H.C.; Huang, C.K.; Gao, H.J.; et al. Detecting multiple lysosomal storage diseases by tandem mass spectrometry - a national newborn screening program in Taiwan. *Clinica Chimica Acta* **2014**, *431*, 80-86, doi:http://dx.doi.org/10.1016/j.cca.2014.01.030.
25. Stapleton, M.; Kubaski, F.; Mason, R.W.; Shintaku, H.; Kobayashi, H.; Yamaguchi, S.; Taketani, T.; Suzuki, Y.; Orii, K.; Orii, T.; et al. Newborn screening for mucopolysaccharidoses: measurement of glycosaminoglycans by LC-MS/MS. *Molecular Genetics and Metabolism Reports* **2020**, *22*, 100563, doi:http://dx.doi.org/10.1016/j.ymgmr.2019.100563.
26. Paciotti, S.; Persichetti, E.; Pagliardini, S.; Deganuto, M.; Rosano, C.; Balducci, C.; Codini, M.; Filocamo, M.; Menghini, A.R.; Pagliardini, V.; et al. First pilot newborn screening for four lysosomal storage diseases in an Italian region: identification and analysis of a putative causative mutation in the GBA gene. *Clinica Chimica Acta* **2012**, *413*, 1827-1831, doi:http://dx.doi.org/10.1016/j.cca.2012.07.011.
27. Chuang, C.K.; Lee, C.L.; Tu, R.Y.; Lo, Y.T.; Sisca, F.; Chang, Y.H.; Liu, M.Y.; Liu, H.Y.; Chen, H.J.; Kao, S.M.; et al. Nationwide newborn screening program for mucopolysaccharidoses in Taiwan and an update of the "gold standard" criteria required to make a confirmatory diagnosis. *Diagnostics (Basel)* **2021**, *11*, 1583, doi:http://dx.doi.org/10.3390/diagnostics11091583.
28. National MPS Society. Advisory Committee on Heritable Disorders in Newborns and Children votes to approve MPS II for Recommended Uniform Screening Panel. https://mpssociety.org/advisory-committee-on-heritable-disorders-in-newborns-and-children-votes-to-approve-mps-ii-for-recommended-uniform-screening-panel/. Available online: https://mpssociety.org/advisory-committee-on-heritable-disorders-in-newborns-and-children-votes-to-approve-mps-ii-for-recommended-uniform-screening-panel/ (accessed on July 1, 2022).

29. Ream, M.A.; Lam, W.K.K.; Grosse, S.D.; Ojodu, J.; Jones, E.; Prosser, L.A.; Rose, A.M.; Comeau, A.M.; Tanksley, S.; Powell, C.M.; et al. Evidence and recommendation for mucopolysaccharidosis type II newborn screening in the United States. *Genet Med* **2023**, *25*, 100330, doi:https://dx.doi.org/10.1016/j.gim.2022.10.012.
30. Millington, D.S.; Ficicioglu, C. Addition of MPS-II to the recommended uniform screening panel in the United States. *Int J Neonatal Screen* **2022**, *8*, doi:https://dx.doi.org/10.3390/ijns8040055.
31. Page, M.J.; McKenzie, J.E.; Bossuyt, P.M.; Boutron, I.; Hoffmann, T.C.; Mulrow, C.D.; Shamseer, L.; Tetzlaff, J.M.; Akl, E.A.; Brennan, S.E.; et al. The PRISMA 2020 statement: an updated guideline for reporting systematic reviews. *Bmj* **2021**, *372*, n71, doi:http://dx.doi.org/10.1136/bmj.n71.
32. National Institute for Health and Care Research. International prospective register of systematic reviews (PROSPERO). https://www.crd.york.ac.uk/prospero/. Available online: https://www.crd.york.ac.uk/prospero/ (accessed on February 21, 2023).
33. Alonso-Fernández, J.R.; Fidalgo, J.; Colón, C. Neonatal screening for mucopolysaccharidoses by determination of glycosaminoglycans in the eluate of urine-impregnated paper: preliminary results of an improved DMB-based procedure. *Journal of Clinical Laboratory Analysis* **2010**, *24*, 149-153, doi:http://dx.doi.org/10.1002/jcla.20375.
34. Bilyeu, H.; Washburn, J.; Vermette, L.; Klug, T. Validation and implementation of a highly sensitive and efficient newborn screening assay for mucopolysaccharidosis type II. *International Journal of Neonatal Screening* **2020**, *6*, 79, doi:https://dx.doi.org/10.3390/ijns6040079.
35. Brower, A.; Chan, K.; Williams, M.; Berry, S.; Currier, R.; Rinaldo, P.; Caggana, M.; Gaviglio, A.; Wilcox, W.; Steiner, R.; et al. Population-based screening of newborns: findings from the NBS Expansion study (Part One). *Front Genet* **2022**, *13*, 867337, doi:https://dx.doi.org/10.3389/fgene.2022.867337.
36. Burton, B.K.; Hoganson, G.E.; Fleischer, J.; Grange, D.K.; Braddock, S.R.; Hickey, R.; Hitchins, L.; Groepper, D.; Christensen, K.M.; Kirby, A.; et al. Population-based newborn screening for mucopolysaccharidosis type II in Illinois: the first year experience. *Journal of Pediatrics* **2019**, *214*, 165-167.e161, doi:http://dx.doi.org/10.1016/j.jpeds.2019.07.053.
37. Burton, B.K.; Hickey, R.; Hitchins, L. Newborn screening for mucopolysaccharidosis type II in Illinois: an update. *International Journal of Neonatal Screening* **2020**, *6*, 73, doi:http://dx.doi.org/10.3390/IJNS6030073.
38. Burton, B.K.; Shively, V.; Quadri, A.; Warn, L.; Burton, J.; Grange, D.K.; Christensen, K.; Groepper, D.; Ashbaugh, L.; Ehrhardt, J.; et al. Newborn screening for mucopolysaccharidosis type II: Lessons learned. *Mol Genet Metab* **2023**, 107557, doi:https://dx.doi.org/10.1016/j.ymgme.2023.107557.
39. Chan, M.J.; Liao, H.C.; Gelb, M.H.; Chuang, C.K.; Liu, M.Y.; Chen, H.J.; Kao, S.M.; Lin, H.Y.; Huang, Y.H.; Kumar, A.B.; et al. Taiwan National Newborn Screening Program by tandem mass spectrometry for mucopolysaccharidoses types I, II, and VI. *Journal of Pediatrics* **2019**, *205*, 176-182, doi:http://dx.doi.org/10.1016/j.jpeds.2018.09.063.
40. Chennamaneni, N.K.; Kumar, A.B.; Barcenas, M.; Spacil, Z.; Scott, C.R.; Turecek, F.; Gelb, M.H. Improved reagents for newborn screening of mucopolysaccharidosis types I, II, and VI by tandem mass spectrometry. *Analytical chemistry* **2014**, *86*, 4508-4514, doi:http://dx.doi.org/10.1021/ac5004135.
41. Chuang, C.K.; Tu, Y.R.; Lee, C.L.; Lo, Y.T.; Chang, Y.H.; Liu, M.Y.; Liu, H.Y.; Chen, H.J.; Kao, S.M.; Wang, L.Y.; et al. Updated confirmatory diagnosis for mucopolysaccharidoses in taiwanese infants and the application of gene variants. *Int J Mol Sci* **2022**, *23*, doi:http://dx/doi.org/10.3390/ijms23179979.
42. Civallero, G.; Michelin, K.; de Mari, J.; Viapiana, M.; Burin, M.; Coelho, J.C.; Giugliani, R. Twelve different enzyme assays on dried-blood filter paper samples for detection of patients with selected inherited lysosomal storage diseases. *Clinica Chimica Acta* **2006**, *372*, 98-102, doi:http://dx.doi.org/10.1016/j.cca.2006.03.029.
43. de Ruijter, J.; de Ru, M.H.; Wagemans, T.; L, I.J.; Lund, A.M.; Orchard, P.J.; Schaefer, G.B.; Wijburg, F.A.; van Vlies, N. Heparan sulfate and dermatan sulfate derived disaccharides are sensitive markers for newborn

screening for mucopolysaccharidoses types I, II and III. *Molecular Genetics and Metabolism* **2012**, *107*, 705-710, doi:http://dx.doi.org/10.1016/j.ymgme.2012.09.024.

44. Fabie, N.A.V.; Pappas, K.B.; Feldman, G.L. The current state of newborn screening in the United States. *Pediatric Clinics of North America* **2019**, *66*, 369-386, doi:http://dx.doi.org/10.1016/j.pcl.2018.12.007.
45. Fdil, N.; Sabir, E.S.; Ezoubeiri, A.; Elqadiry, R.; Daoudi, A.; Lalaoui, A.; Fouad, A.; Rada, N.; Slitine, N.; Bennaoui, F.; et al. Implementation of an affordable method for MPS diagnosis from urine screening to enzymatic confirmation: results of a pilot study in Morocco. *Clin Lab* **2020**, *66*, 3298, doi:http://dx.doi.org/10.7754/Clin.Lab.2019.190720.
46. Gelb, M.H.; Lukacs, Z.; Ranieri, E.; Schielen, P.C.J.I. Newborn screening for lysosomal storage disorders: methodologies for measurement of enzymatic activities in dried blood spots. *International Journal of Neonatal Screening* **2019**, *5*, 1, doi:https://dx.doi.org/10.3390/ijns5010001.
47. Joseph, R.; DiCesare, E.B.; Miller, A. Hunter Syndrome: is it time to make it part of newborn screening? *Advances in neonatal care : official journal of the National Association of Neonatal Nurses* **2018**, *18*, 480-487, doi:http://dx.doi.org/10.1097/ANC.0000000000000569.
48. Jones, S.A.; Cheillan, D.; Chakrapani, A.; Church, H.J.; Heales, S.; Wu, T.H.Y.; Morton, G.; Roberts, P.; Sluys, E.F.; Burlina, A. Application of a novel algorithm for expanding newborn screening for inherited metabolic disorders across Europe. *Int J Neonatal Screen* **2022**, *8*, doi:https://dx.doi.org/10.3390/ijns8010020.
49. Khaledi, H.; Gelb, M.H. Tandem mass spectrometry enzyme assays for multiplex detection of 10-mucopolysaccharidoses in dried blood spots and fibroblasts. *Analytical Chemistry* **2020**, *92*, 11721-11727, doi:https://dx.doi.org/10.1021/acs.analchem.0c01750.
50. Kumar, A.B.; Masi, S.; Ghomashchi, F.; Chennamaneni, N.K.; Ito, M.; Scott, C.R.; Turecek, F.; Gelb, M.H.; Spacil, Z. Tandem mass spectrometry has a larger analytical range than fluorescence assays of lysosomal enzymes: application to newborn screening and diagnosis of mucopolysaccharidoses types II, IVA, and VI. *Clinical Chemistry* **2015**, *61*, 1363-1371, doi:http://dx.doi.org/10.1373/clinchem.2015.242560.
51. Kumar, A.B.; Hong, X.; Yi, F.; Wood, T.; Gelb, M.H. Tandem mass spectrometry-based multiplex assays for α-mannosidosis and fucosidosis. *Molecular Genetics and Metabolism* **2019**, *127*, 207-211, doi:http://dx.doi.org/10.1016/j.ymgme.2019.05.016.
52. Lee, K.; Jun, S.H.; Song, S.H.; Park, H.D.; Park, K.U.; Song, J. Direct assay of iduronate-2-sulfatase for Hunter disease using UPLC-tandem mass spectrometry and fluorogenic substrate. *Clinical Biochemistry* **2015**, *48*, 1350-1353, doi:http://dx.doi.org/10.1016/j.clinbiochem.2015.08.016.
53. Lin, H.Y.; Chang, Y.H.; Lee, C.L.; Tu, Y.R.; Lo, Y.T.; Hung, P.W.; Niu, D.M.; Liu, M.Y.; Liu, H.Y.; Chen, H.J.; et al. Newborn screening program for mucopolysaccharidosis type II and long-term follow-up of the screen-positive subjects in Taiwan. *J Pers Med* **2022**, *12*, 1023, doi:http://dx.doi.org/10.3390/jpm12071023.
54. Lisi, E.C.; McCandless, S.E. Newborn screening for lysosomal storage disorders: views of genetic healthcare providers. *J Genet Couns* **2016**, *25*, 373-384, doi:http://dx.doi.org/10.1007/s10897-015-9879-8.
55. Liu, Y.; Yi, F.; Kumar, A.B.; Chennamaneni, N.K.; Hong, X.; Scott, C.R.; Gelb, M.H.; Turecek, F. Multiplex tandem mass spectrometry enzymatic activity assay for newborn screening of the mucopolysaccharidoses and type 2 neuronal ceroid lipofuscinosis. *Clinical Chemistry* **2017**, *63*, 1118-1126, doi:http://dx.doi.org/10.1373/clinchem.2016.269167.
56. Maccari, F.; Galeotti, F.; Mantovani, V.; Zampini, L.; Padella, L.; Rigon, L.; Concolino, D.; Fiumara, A.; Pascale, E.; Pittala, A.; et al. Composition and structure of glycosaminoglycans in DBS from 2-3-day-old newborns for the diagnosis of mucopolysaccharidosis. *Analytical Biochemistry* **2018**, *557*, 34-41, doi:http://dx.doi.org/10.1016/j.ab.2018.07.007.
57. Meikle, P.J.; Ranieri, E.; Simonsen, H.; Rozaklis, T.; Ramsay, S.L.; Whitfield, P.D.; Fuller, M.; Christensen, E.; Skovby, F.; Hopwood, J.J. Newborn screening for lysosomal storage disorders: clinical evaluation of a two-tier strategy. *Pediatrics* **2004**, *114*, 909-916, doi:http://dx.doi.org/10.1542/peds.2004-0583.

58. Meikle, P.J.; Grasby, D.J.; Dean, C.J.; Lang, D.L.; Bockmann, M.; Whittle, A.M.; Fietz, M.J.; Simonsen, H.; Fuller, M.; Brooks, D.A.; et al. Newborn screening for lysosomal storage disorders. *Molecular Genetics and Metabolism* **2006**, *88*, 307-314, doi:http://dx.doi.org/10.1016/j.ymgme.2006.02.013.
59. Menkovic, I.; Marchand, A.S.; Boutin, M.; Auray-Blais, C. Neonatal mass urine screening approach for early detection of mucopolysaccharidoses by UPLC-MS/MS. *Diagnostics* **2019**, *9*, 195, doi:http://dx.doi.org/10.3390/diagnostics9040195.
60. Oguni, T.; Tomatsu, S.; Tanaka, M.; Orii, K.; Fukao, T.; Watanabe, J.; Fukuda, S.; Notsu, Y.; Vu, D.C.; Can, T.B.N.; et al. Validation of liquid chromatography-tandem mass spectrometry-based 5-plex assay for mucopolysaccharidoses. *International Journal of Molecular Sciences* **2020**, *21*, 2025, doi:http://dx.doi.org/10.3390/ijms21062025.
61. Ruijter, G.J.G.; Goudriaan, D.A.; Boer, A.M.; Van den Bosch, J.; Van der Ploeg, A.T.; Elvers, L.H.; Weinreich, S.S.; Reuser, A.J. Newborn screening for Hunter disease: a small-scale feasibility study. *JIMD rep* **2014**, *14*, 23-27, doi:https://dx.doi.org/10.1007/8904_2013_279.
62. Sabir, E.S.; Lafhal, K.; Ezoubeiri, A.; Harkati, I.; Sbyea, S.; Aldamiz-Echevarria, L.; Andrade, F.; Ait Babram, M.; Maoulainine, F.M.R.; Draiss, G.; et al. Usefulness of urinary glycosaminoglycans assay for a mucopolysaccharidosis-specific screening. *Pediatrics International* **2020**, *62*, 1077-1085, doi:http://dx.doi.org/10.1111/ped.14278.
63. Shimada, T.; Kelly, J.; LaMarr, W.A.; van Vlies, N.; Yasuda, E.; Mason, R.W.; Mackenzie, W.; Kubaski, F.; Giugliani, R.; Chinen, Y.; et al. Novel heparan sulfate assay by using automated high-throughput mass spectrometry: application to monitoring and screening for mucopolysaccharidoses. *Molecular Genetics and Metabolism* **2014**, *113*, 92-99, doi:http://dx.doi.org/10.1016/j.ymgme.2014.07.008.
64. Wang, D.; Wood, T.; Sadilek, M.; Scott, C.R.; Turecek, F.; Gelb, M.H. Tandem mass spectrometry for the direct assay of enzymes in dried blood spots: application to newborn screening for mucopolysaccharidosis II (Hunter disease). *Clinical Chemistry* **2007**, *53*, 137-140, doi:http://dx.doi.org/10.1373/clinchem.2006.077263.
65. Yamaguchi, S. Newborn screening in Japan: restructuring for the new era. *Annals of the Academy of Medicine, Singapore* **2008**, *37*, 13-15.
66. Chien, Y.H.; Hwu, W.L.; Chen, P.W.; Chiu, P.C.; Chu, S.Y.; Yeh, H.Y.; Lee, A.R.; Lee, N.C.; Gelb, M. Newborn screening for Morquio syndrome: results from the 8-plex assay for 70,000 newborns. *Molecular Genetics and Metabolism* **2020**, *129*, S40-S41, doi:http://dx.doi.org/10.1016/j.ymgme.2019.11.082.
67. Chuang, C.K.; Lin, H.Y.; Lo, Y.T.; Tu, R.Y.; Lin, S.P. Status of newborn screening for mucopolysaccharidoses I, II, and VI in Taiwan, including follow up investigations. *Journal of Inherited Metabolic Disease* **2019**, *42* 67, doi:http://dx.doi.org/10.1002/jimd.12153.
68. Gelb, M.H. Newborn screening and post-screening diagnosis of lysosomal diseases. *Molecular Genetics and Metabolism* **2017**, *120*, S50-S51, doi:http://dx.doi.org/10.1016/j.ymgme.2016.11.108.
69. Gelb, M.H. A universal newborn and diagnostic screening platform for lysosomal diseases and beyond. *Molecular Genetics and Metabolism* **2020**, *129*, S61, doi:http://dx.doi.org/10.1016/j.ymgme.2019.11.140.
70. Gelb, M.H. Glycosaminoglycan biomarkers in newborn dried blood spots to support newborn screening of MPS disorders. *Molecular Genetics and Metabolism* **2020**, *129*, S61, doi:http://dx.doi.org/10.1016/j.ymgme.2019.11.141.
71. Matern, D.; Tortorelli, S.; Gavrilov, D.; Raymond, K.; Tang, H.; Lorey, F.; Rinaldo, P.; Oglesbee, D. The (surprising) prevalence of 12 lysosomal storage disorders, Friedreich ataxia, Wilson disease and X-adrenoleukodystrophy in California. *Molecular Genetics and Metabolism* **2014**, *111*, 271, doi:https://doi.org/10.1016/j.ymgme.2014.01.004.
72. Pollard, L.; Wood, T. High prevalence of pseudodeficiency for MPS I and MPS II and the impact on newborn screening. *Molecular Genetics and Metabolism* **2020**, *129*, S130-S131, doi:http://dx.doi.org/10.1016/j.ymgme.2019.11.341.

73. Quadri, A.M.; Kim, K.H.; Hickey, R.E.; Paras, A.; Baker, J.; Charrow, J.; Burton, B.K. Population-based newborn screening for mucopolysaccharidosis type II: a single center's experience. *Molecular Genetics and Metabolism* **2021**, *132*, S89-S90, doi:http://dx.doi.org/10.1016/j.ymgme.2020.12.214.
74. Tomatsu, S.; Kubaski, F.; Mason, R.W. Newborn screening for mucopolysaccharidoses by GAG assay with tandem mass spectrometry. *Molecular Genetics and Metabolism* **2019**, *126*, S145, doi:https://doi.org/10.1016/j.ymgme.2018.12.374.
75. Ullal, A.; Ng, R.; Nuffer, M.; Graham, C.; Nelson, L.; Singh, R.; Pamula, V. Flexible digital microfluidic platform to multiplex various combinations of enzymatic assays for newborn screening of Pompe, mucopolysaccharidosis types I and II, biotinidase deficiency and galactosemia disorders. *Molecular Genetics and Metabolism* **2017**, *120*, S132-S133, doi:http://dx.doi.org/10.1016/j.ymgme.2016.11.349.
76. Van Vlies, N.; De Ruijter, J.; De Ru, M.H.; Wagemans, T.; Ijlst, L.; Wijburg, F.A. Heparan sulfate and dermatan sulfate disaccharide levels for newborn screening in MPS I, MPS II and MPS III. *Molecular Genetics and Metabolism* **2013**, *108*, S95, doi:http://dx.doi.org/10.1016/j.ymgme.2012.11.259.
77. Wasserstein, M.; Caggana, M.; Gelb, M.H.; Goldenberg, A.; Kelly, N.; Matern, D.; Orsini, J.J. ScreenPlus: a comprehensive, dynamic, multi-disorder newborn screening pilot program. *Molecular Genetics and Metabolism* **2020**, *129*, S160, doi:http://dx.doi.org/10.1016/j.ymgme.2019.11.426.
78. Pique, D. A novel visualization of state-by-state variability in newborn screening highlights underrepresentation of lysosomal storage disorders. *Genet Med* **2022**, *24*, S321-S322; abstract #eP504.
79. Okuyama, T.; Seo, J.H.; Ohtake, A. Current status of newborn screening for lysosomal diseases in Japan: Importance of novel therapies for central nervous system manifestation in MPS II, and importance of family screening of Fabry disease after newborn screening. *Mol Genet Metab* **2022**, *135*, S15-S132; abstract #219.
80. Taiwan Ministry of Health and Welfare Health Promotion Administration. The first line of defense for health: three decades of newborn screening in Taiwan (2016). Available online: https://www.hpa.gov.tw/Pages/ashx/File.ashx?FilePath=~/File/Attach/6471/File_6142.pdf (accessed on February 20, 2023).
81. World Population Review. Birth rate by country 2022. https://worldpopulationreview.com/country-rankings/birth-rate-by-country. Available online: https://worldpopulationreview.com/country-rankings/birth-rate-by-country (accessed on February 21, 2023).
82. Center for Disease Control and Prevention National Center for Health Statistics. Life stages and population: State and territorial data. https://www.cdc.gov/nchs/fastats/state-and-territorial-data.htm. Available online: https://www.cdc.gov/nchs/fastats/state-and-territorial-data.htm (accessed on February 21, 2023).
83. Health, I.D.o.P. Birth Statistics. https://dph.illinois.gov/data-statistics/vital-statistics/birth-statistics.html. Available online: https://dph.illinois.gov/data-statistics/vital-statistics/birth-statistics.html (accessed on May 1, 2024).
84. MedLine Plus. Mucopolysaccharidosis type II. Available online: https://medlineplus.gov/genetics/condition/mucopolysaccharidosis-type-ii/ (accessed on April 16, 2024).
85. National MPS Society. MPS II (Hunter Syndrome). https://mpssociety.org/learn-about-mps/diseases/mps-ii/. Available online: https://mpssociety.org/learn-about-mps/diseases/mps-ii/ (accessed on April 16, 2024).
86. Pamula, V.; Sista, R.; Wang, T.; Graham, C.; Eckhardt, A.; Winger, T.; Wu, N.; Xiong, Y.; Srinivasan, V.; Millington, D.; et al. Digital microfluidic platform for multiplexing LSD assays in newborn screening. *Molecular Genetics and Metabolism* **2011**, *102*, S33, doi:http://dx.doi.org/10.1016/j.ymgme.2010.11.112.
87. Sista, R.; Eckhardt, A.E.; Wang, T.; Séllos-Moura, M.; Pamula, V.K. Rapid, single-step assay for Hunter syndrome in dried blood spots using digital microfluidics. *Clinica Chimica Acta* **2011**, *412*, 1895-1897, doi:https://doi.org/10.1016/j.cca.2011.06.015.

88. Pamula, V.; Sista, R.; Wang, T.; Graham, C.; Wu, N.; Eckhardt, A.; Winger, T.; Xiong, Y.; Millington, D.; Ali, D. Rapid LSD assays on a multiplex digital microfluidic platform for newborn screening. *Molecular Genetics and Metabolism* **2012**, *105*, S49, doi:http://dx.doi.org/10.1016/j.ymgme.2011.11.124.
89. Monachesi, C.; Zampini, L.; Padella, L.; Marchesiello, R.L.; Galeazzi, T.; Santoro, L.; Catassi, C.; Gasparrini, E.; Carnielli, V.P.; Volpi, N.; et al. False positive screen test for mucopolysaccharidoses in healthy female newborns. *Clinica Chimica Acta* **2018**, *486*, 221-223, doi:http://dx.doi.org/10.1016/j.cca.2018.08.016.
90. Burlina, A.; Jones, S.A.; Chakrapani, A.; Church, H.J.; Heales, S.; Wu, T.H.Y.; Morton, G.; Roberts, P.; Sluys, E.F.; Cheillan, D. A new approach to objectively evaluate inherited metabolic diseases for inclusion on newborn screening programmes. *Int J Neonatal Screen* **2022**, *8*, doi:https://dx.doi.org/10.3390/ijns8020025.
91. Olney, R.S.; Bonham, J.R.; Schielen, P.C.J.I.; Slavin, D.; Ojodu, J. 2023 APHL/ISNS Newborn Screening Symposium. *International Journal of Neonatal Screening* **2023**, *9*, 54.
92. Maternal and Child Health Bureau of the Health and Resources and Services Administration. Newborn screening for mucopolysaccharidosis type I. a summary of the evidence and advisory committee decision .https://www.hrsa.gov/sites/default/files/hrsa/advisory-committees/heritable-disorders/mps-i-27-june-2018.pdf. Available online: https://www.hrsa.gov/sites/default/files/hrsa/advisory-committees/heritable-disorders/mps-i-27-june-2018.pdf (accessed on July 1, 2022).
93. Puckett, Y.; Mallorga-Hernandez, A.; Montano, A.M. Epidemiology of mucopolysaccharidoses (MPS) in United States: challenges and opportunities. *Orphanet J Rare Dis* **2021**, *16*, 241, doi:http://dx.doi.org/10.1186/s13023-021-01880-8.
94. Association of Public Health Laboratories. Newborn screening status for all disorders. https://www.newsteps.org/resources/data-visualizations/newborn-screening-status-all-disorders. Available online: https://www.newsteps.org/resources/data-visualizations/newborn-screening-status-all-disorders (accessed on November 1, 2023).
95. Wraith, J.E.; Beck, M.; Giugliani, R.; Clarke, J.; Martin, R.; Muenzer, J. Initial report from the Hunter Outcome Survey. *Genet Med* **2008**, *10*, 508-516, doi:https://dx.doi.org/10.1097/gim.0b013e31817701e6.
96. National Organization for Rare Disorders (NORD). Mucopolysaccharidosis type II. https://rarediseases.org/rare-diseases/mucopolysaccharidosis-type-ii-2/. Available online: https://rarediseases.org/rare-diseases/mucopolysaccharidosis-type-ii-2/ (accessed on October 20, 2023).
97. Chen, X.; Qiu, W.; Ye, J.; Han, L.; Gu, X.; Zhang, H. Demographic characteristics and distribution of lysosomal storage disorder subtypes in Eastern China. *Journal of Human Genetics* **2016**, *61*, 345-349, doi:https://dx.doi.org/10.1038/jhg.2015.155.
98. Lin, S.-P.; Chang, J.-H.; Lee-Chen, G.-J.; Lin, D.-S.; Lin, H.-Y.; Chuang, C.-K. Detection of hunter syndrome (mucopolysaccharidosis type II) in Taiwanese: Biochemical and linkage studies of the iduronate-2-sulfatase gene defects in MPS II patients and carriers. *Clinica Chimica Acta* **2006**, *369*, 29-34, doi:https://doi.org/10.1016/j.cca.2006.01.001.
99. Isogai, K.; Sukegawa, K.; Tomatsu, S.; Fukao, T.; Song, X.-Q.; Yamada, Y.; Fukuda, S.; Orii, T.; Kondo, N. Mutation analysis in the iduronate-2-sulphatase gene in 43 Japanese patients with mucopolysaccharidosis type II (Hunter disease). *Journal of Inherited Metabolic Disease* **1998**, *21*, 60-70, doi:https://doi.org/10.1023/A:1005363414792.
100. Millington, D.; Norton, S.; Singh, R.; Sista, R.; Srinivasan, V.; Pamula, V. Digital microfluidics comes of age: high-throughput screening to bedside diagnostic testing for genetic disorders in newborns. *Expert Rev Mol Diagn* **2018**, *18*, 701-712, doi:https://dx.doi.org/10.1080/14737159.2018.1495076.
101. Gelb, M.H. Newborn screening for lysosomal storage diseases: methodologies, screen positive rates, normalization of datasets, second-tier tests, and post-analysis tools. *Int J Neonatal Screen* **2018**, *4*, doi:https://dx.doi.org/10.3390/ijns4030023.

102. Herbst, Z.M.; Urdaneta, L.; Klein, T.; Burton, B.K.; Basheeruddin, K.; Liao, H.C.; Fuller, M.; Gelb, M.H. Evaluation of two methods for quantification of glycosaminoglycan biomarkers in newborn dried blood spots from patients with severe and attenuated mucopolysaccharidosis type II. *Int J Neonatal Screen* **2022**, *8*, doi:https://dx.doi.org/10.3390/ijns8010009.
103. Saville, J.T.; McDermott, B.K.; Fletcher, J.M.; Fuller, M. Disease and subtype specific signatures enable precise diagnosis of the mucopolysaccharidoses. *Genetics in Medicine* **2019**, *21*, 753-757, doi:https://doi.org/10.1038/s41436-018-0136-z.
104. Arunkumar, N.; Vu, D.C.; Khan, S.; Kobayashi, H.; Ngoc Can, T.B.; Oguni, T.; Watanabe, J.; Tanaka, M.; Yamaguchi, S.; Taketani, T.; et al. Diagnosis of mucopolysaccharidoses and mucolipidosis by assaying multiplex enzymes and glycosaminoglycans. *Diagnostics (Basel)* **2021**, *11*, 1347, doi:http://dx.doi.org/10.3390/diagnostics11081347.
105. Polo, G.; Gueraldi, D.; Giuliani, A.; Rubert, L.; Cazzorla, C.; Salviati, L.; Marzollo, A.; Biffi, A.; Burlina, A.P.; Burlina, A.B. The combined use of enzyme activity and metabolite assays as a strategy for newborn screening of mucopolysaccharidosis type I. *Clin Chem Lab Med* **2020**, *58*, 2063-2072, doi:http://dx.doi.org/10.1515/cclm-2020-0064.
106. Ficicioglu, C.; Giugliani, R.; Harmatz, P.; Mendelsohn, N.J.; Jego, V.; Parini, R. Intrafamilial variability in the clinical manifestations of mucopolysaccharidosis type II: data from the Hunter Outcome Survey (HOS). *Am J Med Genet A* **2018**, *176*, 301-310, doi:http://dx.doi.org/10.1002/ajmg.a.38551.
107. Josahkian, J.A.; Brusius-Facchin, A.C.; Netto, A.B.O.; Leistner-Segal, S.; Malaga, D.R.; Burin, M.G.; Michelin-Tirelli, K.; Trapp, F.B.; Cardoso-Dos-Santos, A.C.; Ribeiro, E.M.; et al. Genotype–phenotype studies in a large cohort of Brazilian patients with Hunter syndrome. *Am J Med Genet C Semin Med Genet* **2021**, *187*, 349-356, doi:http://dx.doi.org/10.1002/ajmg.c.31915.
108. Vollebregt, A.A.M.; Hoogeveen-Westerveld, M.; Kroos, M.A.; Oussoren, E.; Plug, I.; Ruijter, G.J.; van der Ploeg, A.T.; Pijnappel, W. Genotype–phenotype relationship in mucopolysaccharidosis II: predictive power of *IDS* variants for the neuronopathic phenotype. *Dev Med Child Neurol* **2017**, *59*, 1063-1070, doi:http://dx.doi.org/10.1111/dmcn.13467.
109. Friedman, J.M.; Cornel, M.C.; Goldenberg, A.J.; Lister, K.J.; Senecal, K.; Vears, D.F.; Global Alliance for, G.; Health, R.; Ethics Working Group Paediatric Task, T. Genomic newborn screening: public health policy considerations and recommendations. *BMC Med Genomics* **2017**, *10*, 9, doi:http://dx.doi.org/10.1186/s12920-017-0247-4.
110. Johnston, J.; Lantos, J.D.; Goldenberg, A.; Chen, F.; Parens, E.; Koenig, B.A.; members of the, N.E.; Policy Advisory, B. Sequencing newborns: a call for nuanced use of genomic technologies. *Hastings Cent Rep* **2018**, *48*, S2-S6, doi:http://dx.doi.org/10.1002/hast.874.
111. Bick, D.; Ahmed, A.; Deen, D.; Ferlini, A.; Garnier, N.; Kasperaviciute, D.; Leblond, M.; Pichini, A.; Rendon, A.; Satija, A.; et al. Newborn screening by genomic sequencing: opportunities and challenges. *Int J Neonatal Screen* **2022**, *8*, 40, doi:http://dx.doi.org/10.3390/ijns8030040.
112. Kanungo, S.; Patel, D.R.; Neelakantan, M.; Ryali, B. Newborn screening and changing face of inborn errors of metabolism in the United States. *Ann Transl Med* **2018**, *6*, 468, doi:http://dx.doi.org/10.21037/atm.2018.11.68.
113. Lin, H.Y.; Lee, C.L.; Chang, C.Y.; Chiu, P.C.; Chien, Y.H.; Niu, D.M.; Tsai, F.J.; Hwu, W.L.; Lin, S.J.; Lin, J.L.; et al. Survival and diagnostic age of 175 Taiwanese patients with mucopolysaccharidoses (1985–2019). *Orphanet J Rare Dis* **2020**, *15*, 314, doi:http://dx.doi.org/10.1186/s13023-020-01598-z.
114. Blackwell, K.; Gelb, M.H.; Grantham, A.; Spencer, N.; Webb, C.; West, T. Family attitudes regarding newbornscreening for Krabbe Disease: results from a survey of leukodystrophy registries. *International Journal of Neonatal Screening* **2020**, *6*, 66, doi:http://dx.doi.org/10.3390/IJNS6030066.

115. Bouwman, M.G.; de Ru, M.H.; Linthorst, G.E.; Hollak, C.E.M.; Wijburg, F.A.; van Zwieten, M.C.B. Fabry patients' experiences with the timing of diagnosis relevant for the discussion on newborn screening. *Molecular Genetics and Metabolism* **2013**, *109*, 201-207, doi:http://dx.doi.org/10.1016/j.ymgme.2013.03.008.
116. DeLuca, J.M. Public attitudes toward expanded newborn screening. *Journal of Pediatric Nursing* **2018**, *38*, e19-e23, doi:http://dx.doi.org/10.1016/j.pedn.2017.10.002.
117. Tao-Nishida, E.; Seo, J.H.; Sohn, Y.B.; Yotsumoto, J.; Kosuga, M.; Tanaka, T.; Omori, M.; Kawame, H.; Jin, D.K.; Okuyama, T. What do you think of enzyme replacement therapy and newborn screening for mucopolysaccharidoses? Opinions from patients and families of patients in Japan and Korea. *Journal of Inherited Metabolic Disease* **2010**, *33*, S128, doi:http://dx.doi.org/10.1007/s10545-010-9163-x.
118. Hayes, I.M.; Collins, V.; Sahhar, M.; Wraith, J.E.; Delatycki, M.B. Newborn screening for mucopolysaccharidoses: opinions of patients and their families. *Clinical Genetics* **2007**, *71*, 446-450, doi:http://dx.doi.org/10.1111/j.1399-0004.2007.00783.x.
119. Lisi, E.C.; Ali, N. Opinions of adults affected with later-onset lysosomal storage diseases regarding newborn screening: A qualitative study. *J Genet Couns* **2021**, *30*, 1544-1558, doi:https://dx.doi.org/10.1002/jgc4.1421.
120. Lisi, E.C.; Gillespie, S.; Laney, D.; Ali, N. Patients' perspectives on newborn screening for later-onset lysosomal storage diseases. *Molecular Genetics and Metabolism* **2016**, *119*, 109-114, doi:http://dx.doi.org/10.1016/j.ymgme.2016.07.009.
121. Lisi, E.C.; Long, V.; Laney, D. Do the benefits outweigh the harms? Views of patients with later onset LSD on newborn screening. *Molecular Genetics and Metabolism* **2015**, *114*, S74, doi:http://dx.doi.org/10.1016/j.ymgme.2014.12.159.
122. Peterson, L.; Siemon, A.; Olewiler, L.; McBride, K.L.; Allain, D.C. A qualitative assessment of parental experiences with false-positive newborn screening for Krabbe disease. *J Genet Couns* **2022**, *31*, 252-260, doi:http://dx.doi.org/10.1002/jgc4.1480.
123. Hall, J.G. The role of patient advocacy/parent support groups. *S Afr Med J* **2013**, *103*, 1020-1022, doi:http://dx.doi.org/10.7196/samj.6976.
124. Nicholl, H.; Tracey, C.; Begley, T.; King, C.; Lynch, A.M. Internet use by parents of children with rare conditions: findings from a study on parents' web information needs. *J Med Internet Res* **2017**, *19*, e51, doi:http://dx.doi.org/10.2196/jmir.5834.
